# Long-term administration of intravenous Trappsol® Cyclo™ (HP-β-CD) results in clinical benefits and stabilization or slowing of disease progression in patients with Niemann-Pick disease type C1: Results of an international 48-week Phase I/II trial

**DOI:** 10.1016/j.ymgmr.2023.100988

**Published:** 2023-06-29

**Authors:** Reena Sharma, Caroline Hastings, Orna Staretz-Chacham, Julian Raiman, Martin Paucar, Ronen Spiegel, Bryan Murray, Bryan Hurst, Benny Liu, Lise Kjems, Sharon Hrynkow

**Affiliations:** a Salford Royal Hospital NHS Foundation Trust, Department of Adult Inherited Metabolic Diseases, Stott Lane, Salford, Greater Manchester M6 8HD, UK; b UCSF Benioff Children's Hospital, Oakland, CA 94609, USA; c Soroka Medical Center, Rager Blvd, P.O.B. 151, Beer Sheva 84101, Israel; d Birmingham Children's Hospital, Steelhouse Lane, Birmingham B4 6NH, UK; e Karolinska University Hospital, Huddinge, Department of Neurology, R43 Rehabgatan, 4th Floor, 141 86 Stockholm, Sweden; f Department of Pediatrics B, Emek Medical Center, Afula 1834111, Israel; g The Ruth and Bruce Rappaport Faculty of Medicine, Technion, Haifa, Israel; h Boyd Consultants Ltd, Electra House, Crewe Business Park, Crewe, Cheshire CW1 6GL, UK; i Highland Hospital, 1411 East 31st Street, Oakland, CA 94602, USA; j Cyclo Therapeutics, Inc, 6714 NW 16th Street, Suite B, Gainesville, FL 32653, USA

**Keywords:** Cyclodextrin, Cholesterol homeostasis, Niemann-Pick disease type C, Biomarker, Disease stabilization, NPC clinical severity scale

## Abstract

**Background:**

Niemann-Pick disease type C (NPC) is a rare, fatal, pan-ethnic, autosomal recessive lysosomal storage disease characterized by progressive major organ failure and neurodegeneration. Preclinical studies confirmed a critical role of systemically administered hydroxypropyl-β-cyclodextrin (HP-β-CD; Trappsol**®** Cyclo™) in cholesterol metabolism and homeostasis in peripheral tissues of the body, including the liver, and in the central nervous system (CNS). Herein, the pharmacokinetics (PK), safety, and efficacy of HP-β-CD, and biomarkers of NPC were assessed in pediatric and adult patients with NPC1.

**Methods:**

This was a multicenter, Phase I/II, randomized, double-blind, parallel-group, 48-week study (ClinicalTrials.gov identifier NCT02912793) to compare the PK of three different single intravenous (IV) doses of HP-β-CD in pediatric and adult patients with NPC1 and to evaluate the efficacy and tolerability of three different dosages of HP-β-CD in patients with NPC1 after long-term treatment. Twelve patients aged at least 2 years (2–39 years of age) with a confirmed diagnosis of NPC1 were randomized to receive one of three IV doses of HP-β-CD (1500 mg/kg, 2000 mg/kg, or 2500 mg/kg) every 2 weeks for 48 weeks. All patients received HP-β-CD; there was no placebo or other control. PK testing of plasma and cerebrospinal fluid (CSF) was at set times after the first infusion. Pharmacodynamic assessments included biomarkers of cholesterol metabolism (synthesis and breakdown products), *N*-palmitoyl-*O*-phosphocholineserine (PPCS), and specific biomarkers of CSF neurodegeneration (including total Tau), CNS inflammation (glial fibrillary acidic protein [GFAP] and tumor necrosis factor α [TNFα]), CNS cholesterol metabolism (24S-hydroxycholesterol) and inflammatory markers. Efficacy measures included clinical disease severity, neurologic symptoms, and clinical impressions of improvement. Safety assessment included physical examination, vital signs, clinical safety laboratory assessment and adverse events (AEs).

**Results:**

Nine patients completed the study, 2 in the 1500 mg/kg group, 4 in the 2000 mg/kg group and 3 in the 2500 mg/kg group. Three patients (all in the 1500 mg/kg group) discontinued the study because of either physician decision/site Principal Investigator (PI) discretion, withdrawal by subject/patient/parent/guardian, or other non-safety reasons. In 5 patients who underwent serial lumbar punctures, HP-β-CD was detected in the CSF. Of the 9 patients who completed the study, 8 (88.9%) improved in at least two domains of the 17-Domain Niemann-Pick disease Type C-Clinical Severity Scale (17D-NPC-CSS), and 6 of these patients improved in at least one domain viewed by patients and their caregivers to be key to quality of life, namely, speech, swallow, fine and gross motor skills, and cognition. Of the 9 patients who completed the study, 7 were viewed by their treating physicians as having improved to some degree at the end of the study, and 2 remained stable; both outcomes are highly relevant in a progressive neurodegenerative disease. Some patients and families reported improvement in quality of life.

All three doses of HP-β-CD were well tolerated overall, with most treatment-emergent adverse events transient, mild-to-moderate in nature, and considered by the site PIs to be not related to study drug.

**Interpretation:**

This 48-week trial is the longest to date to evaluate the safety, tolerability, and efficacy across multiple clinical endpoints of IV administration of Trappsol**®** Cyclo™ (HP-β-CD) in NPC1 patients. In pediatric and adult patients with NPC, Trappsol**®** Cyclo™ IV improved clinical signs and symptoms and was generally well tolerated. The findings presented here demonstrate a favorable benefit-risk profile and support the global pivotal trial now underway to evaluate the long-term treatment benefits and the potential of Trappsol**®** Cyclo™ as a disease-modifying treatment in this patient population.

## 1 Introduction

Niemann-Pick Disease Type C (NPC) is a rare, fatal, pan-ethnic, autosomal recessive lysosomal storage disease characterized by progressive major organ failure and neurodegeneration and has an estimated worldwide incidence of 1 in 90,000 to 100,000 live births [[1], [2], [3], [4]]. Molecular genetic testing has indicated that the majority of patients with NPC have disease attributable to loss of function mutations in the *NPC1* (90–95% of patients) or *NPC2* (4% of patients) genes, resulting in impaired intracellular lipid trafficking [[4], [5], [6], [7], [8], [9], [10], [11]], with inflammation, impaired cell functioning, mitochondrial dysfunction and ultimately cell death. The underlying pathological mechanisms in NPC are not fully understood; however, accumulation of lipids in the late endosomes/lysosomes is probably a crucial event in disease pathogenesis [4,12]. The disease is characterized histologically by intracellular protein aggregates (neurofibrillary tangles) and a well-defined pattern of loss of cerebellar Purkinje cells [13,14].

NPC shows an extreme clinical heterogeneity, with symptoms varying in severity and with age of onset. Symptoms associated with NPC include visceral manifestations (such as hepatomegaly, splenomegaly, and lung dysfunction), neurological manifestations (including cerebellar ataxia, dystonia, dysmetria, dysarthria, dysphagia, and vertical supranuclear gaze palsy), cognitive impairment, and psychiatric symptoms [8,9,15]. Although the majority of patients with NPC die aged between 10 and 25 years, the disease may present at any age, from the perinatal period to the seventh decade of life [1,4,8,9,[16], [17], [18], [19], [20]].

A range of therapeutic strategies has been evaluated in preclinical and clinical studies [9]; however, there are currently no treatments approved for NPC that effectively treat both systemic and neurological manifestations of the disease [19], except for symptom management and palliative approaches [1,19].

Miglustat (Zavesca®) is an inhibitor of glucosylceramide synthase, an enzyme involved in the production of glycosphingolipids, and is approved for NPC in the European Union (EU) but not in the US, where it is used off-label [19,21]. This drug has been shown to have beneficial effects on lipid trafficking defects, thus slowing the progression of the neurological symptoms of NPC in some patients, and increasing median survival from time of onset of neurological manifestations by approximately 10 years [19,22]. However, miglustat has no effect on the systemic manifestations of the disease and some patients experience adverse events (AEs) that may complicate, or even prohibit, long-term use of this drug in individual cases, such as persistent and significant diarrhea (various degrees of diarrhea are experienced in up to 80% of patients taking the drug, according to the summary of product characteristics [SmPC] [23]) and persistent tremor that does not improve spontaneously or upon transient dose reduction [24,25]. Therefore, there is an urgent medical need for new and effective treatments for NPC that delay progression and improve quality of life and survival.

Accumulation of lipids such as cholesterol in the endosomes/lysosomes of the cells in patients with NPC1 is regarded as the major contributor to the pathogenicity of this disease and derived clinical signs and symptoms [4,12]. The role of other factors, including changes in cholesterol homeostasis resulting from inappropriate storage in the cells, is much less clear. Therefore, release of trapped sterols should result in less cell/organ damage due to removal of the primary insult and lead to improvement in the metabolic milieu and cell functionality. There is a body of evidence that cyclodextrins like HP-β-CD remove sterols from isolated cells or in animal models of NPC1 [29]. This clinical trial is the second in a series of studies to produce data that support the development of IV HP-β-CD as a novel treatment for NPC1 that targets both the neurological and the systemic clinical signs and symptoms of the disease.

Hydroxypropyl-β-cyclodextrin (HP-β-CD [Trappsol**®** Cyclo™]) is a modified version of a naturally occurring cyclodextrin with a hydrophilic exterior and a hydrophobic core, which enables it to form complexes with hydrophobic compounds and to be used as a delivery vehicle to improve solubility, stability, and bioavailability of various medicinal products [26,27]. Data from in vivo preclinical animal studies, the majority completed using *NPC*-knockout mice, show that HP-β-CD (commercial grade) releases trapped cholesterol in a dose-dependent manner and has several beneficial effects on cholesterol metabolism, delaying the clinical onset of NPC disease, stimulating the movement of cholesterol from the late endosomes/lysosomes to the cytosol in many organs, and ameliorating hepatosplenomegaly and neurological symptoms. The decreasing cholesterol synthesis rates in these studies are a signal that indicates release of free cholesterol from the late endosomes/lysosomes into the cytosol when HP-β-CD is administered. Prolongation of life was also observed in the treated NPC mice and NPC cats [14,[28], [29], [30], [31], [32], [33]].

The body responds to a rise in cholesterol levels in the blood with a negative feedback to reduce the rate of synthesis of cholesterol and increase the rate of catabolism. At the cellular level, once unesterified cholesterol is released from lysosomes corresponding to HP-β-CD administration, cytosolic sensors, including sterol regulatory element-binding protein (SREBP), transmit signals to the nucleus as part of this negative feedback loop [29]. Serum lathosterol concentration is reported to be an indicator of whole-body cholesterol synthesis in man [34] and was measured, along with the cholesterol precursors lanosterol and desmosterol, in the present study.

In addition to these biomarkers of target engagement, disease-related biomarkers were evaluated in this study.

## 2 Materials and methods

### 2.1 Study design

This was a multicenter, Phase I/II, randomized, double-blind, parallel-group study to compare the pharmacokinetics (PK) of three different single IV doses of HP-β-CD in patients with NPC1 and to evaluate the efficacy and tolerability of the three different dosages of HP-β-CD in patients with NPC1 after 48 weeks of treatment. All patients received HP-β-CD; there was no placebo or other control. Further objectives were to investigate the effect of the three doses upon serum and lymphocytic markers of cholesterol metabolism and to evaluate HP-β-CD concentrations in CSF.

More specifically, in Stage 1 of the study (baseline to 20 h post the start of first infusion of study drug), PK in plasma and CSF were evaluated along with serum and lymphocytic markers of cholesterol metabolism (described below). In Stage 2 of the study, beginning at Day 2 of the study and continuing to end of study, evaluation of the study drug on clinical manifestations (neurologic and systemic) were evaluated along with continued evaluation of serum and lymphocytic markers of cholesterol metabolism (described below).

Patients aged at least 2 years with a confirmed diagnosis of NPC1 were enrolled at five centers: two in the UK (Salford Royal Hospital NHS Foundation Trust; Birmingham Children's Hospital), one in Sweden (Karolinska University Hospital, Stockholm), and two in Israel (Soroka Medical Center, Beer Sheva; Emek Medical Center, Afula). Patients in the 2–5 years age range were only admitted after acceptable safety and tolerability had been demonstrated in a cohort of 6 older patients (aged 5+ years), as judged by an independent Safety Review Committee (SRC).

NPC1 diagnosis was defined by one of the following: two *NPC1* mutations on genotyping; one *NPC1* mutation and positive filipin staining (current or prior); vertical supranuclear gaze palsy plus either one or more *NPC1* mutations or positive filipin staining and no *NPC2* mutations.

Patients were assessed for clinical severity based on a cumulative score using the 17-Domain Niemann-Pick disease Type C-Clinical Severity Scale (17D-NPC-CSS), which was developed at the US National Institutes of Health (NIH), and could not score above 30, with no more than 4 individual major domains scoring ≥3 (maximum score in each domain, 5) [35].

Female patients of childbearing age had a negative pregnancy test prior to treatment. Patients with *NPC2* mutations were excluded, as were patients with Stage 3 chronic kidney disease, evidence of acute liver disease, or weight > 100 kg.

Informed consent was obtained from each patient and/or the patient's legally authorized representative, as appropriate, before initiation of treatment or performance of any study-specific screening tests or evaluations in accordance with the Independent Ethics Committee/Institutional Review Board and the principles of ethical research according to the Declaration of Helsinki.

Patients received IV infusions of Trappsol**®** Cyclo™ HP-β-CD, a proprietary formulation of Cyclo Therapeutics, Inc., at doses of 1500 mg/kg, 2000 mg/kg, or 2500 mg/kg body weight over 8–9 h every 2 weeks for a 48-week period, with a follow-up evaluation 28 days after their last study visit. The dose of HP-β-CD infused was made up to a total volume of 250 mL for patients weighing <25 kg, 500 mL for patients weighing ≥25 kg and < 50 kg, or 1000 mL for patients weighing ≥50 kg using normal saline. The patient, the physician, and the study center personnel were blinded to the actual dose the patient received.

Lumbar punctures were performed, either by a temporary catheter or intermittent punctures, to enable serial measurements to obtain CSF concentrations of HP-β-CD measured at 4, 8 and 12 h following the start of the IV administration.

Safety data, including AEs and clinical safety laboratory data, were reviewed by the SRC at periodic intervals per the SRC charter.

### 2.2 Pharmacokinetics

Blood samples were collected to determine plasma drug concentration and to evaluate the time to maximum concentration (t_max_), maximum observed plasma concentration (C_max_), volume of distribution (Vd), clearance (CL), area-under-the-curve from zero to infinity (AUC_0-∞_), and elimination half-life (t_1/2_) after the first dose of HP-β-CD. Samples were collected at 0, 2, 4, 6 and 8 h after the start of the infusion. Further samples were taken at the end of the infusion, and at 0.5, 1, 2, 4, 8, and 12 h later (i.e., approximately 20 h after the start of the infusion).

All samples were analyzed by liquid chromatography-tandem mass spectrometry (LC-MS/MS; Medpace Bioanalytical Laboratories [MBL], Cincinnati, OH, USA).

### 2.3 Pharmacodynamics

Blood samples were taken to measure serum cholesterol precursors (lanosterol, lathosterol, desmosterol) and cholesterol metabolites/bile acid precursors (4β-, 24S-, 25-, 27-hydroxycholesterol), thereby providing a means to assess the effect of HP-β-CD on the overall metabolism of cholesterol. Patients were advised to avoid high cholesterol diets and to fast for 8 h before sampling.

Samples were taken at baseline and Day 1 before the first infusion, with the average of these values forming the baseline. Further samples were taken on Day 2, and at 3, 5, 8 and 15 days post-initial dose, at 4, 6, 8, 10, 12, 16, 20, 24, 28, 32, 36, 40, 44 and 48 weeks (Stage 2 of the study), and at the follow-up visit. Precursors and metabolites were assayed by MBL using gas chromatography–mass spectrometry (GC–MS). *N*-palmitoyl-*O*-phosphocholineserine (PPCS) was measured in plasma by CENTOGENE AG, Am Strande, 18,055 Rostock, Germany using a commercially available, validated LC/MS method.

Lysotracker (Thermo Fisher Scientific) measures the relative acidic compartment volume of peripheral blood mononuclear cells (PBMCs) and this is a validated indicator of disease progression in lysosome storage disorders [36]. In this study, the lysotracker assay was conducted at the Department of Pharmacology, University of Oxford, UK, with samples drawn at baseline, and weeks 2, 12, 24, 36, and 48.

CSF samples for the determination of Tau, TNFα and GFAP were taken by lumbar puncture or catheter (as per local practice/site Principal Investigator (PI) preference) prior to the initiation of the first dose (baseline, Stage 1) and on an optional basis during Stage 2 at Week 24 and Week 48. Samples were analyzed by immunoassay by MBL.

Liver and spleen size were determined using abdominal ultrasound. Reports were read and interpreted by a central radiologist who used standardized methods to measure the longest axis of both liver and spleen and reference measures for healthy subjects to determine hepatosplenomegaly [37,38].

### 2.4 Efficacy outcome measures

Disease-specific assessment tools were used to measure disease progression and clinical severity. Patients were assessed at baseline and weeks 12, 24, 36, and 48 using the 17D-NPC-CSS, which measures 9 major neurologic features of NPC (eye movement, ambulation, speech, swallow, fine motor skills, cognition, hearing [sensorineural], memory, and seizures) and 8 modifying features (gelastic cataplexy, narcolepsy, behavior, psychiatric, hyperreflexia, incontinence, auditory brainstem response [ABR], and respiratory) [35]. Total scores as well as individual domain scores were captured and compared. Additional relevant tools were applied for assessments of neurologic symptoms included the Scale for the Assessment and Rating of Ataxia (SARA) and bead-threading [39], which were also conducted at 12, 24, 36 and 48 weeks. Clinical impressions of improvement (CGI—I; low scores represent much improvement) [40] were measured on a standard 7-point ranking system at 12-week intervals following baseline scoring by clinicians of clinical severity (CGI—S). The Patient Global Impression of Change (PGIC) scale [41] was used at 12, 24, 36 and 48 weeks to evaluate how the patient/their caregiver would describe the change (if any) to the patient's overall health since starting the study, from ‘no change (or worse)’ (the lowest score) to ‘a great deal better and a considerable improvement that has made all the difference’ (the highest score). In addition, the Pediatric Quality of Life survey (PedsQL™) was administered at 12, 24, 36 and 48 weeks.

### 2.5 Safety outcome measures

Blood was collected to assess a standard panel of clinical chemistry and hematology laboratory parameters to monitor safety. Clinical serum chemistry analyses included urea and electrolytes, hepatic transaminases, bilirubin, serum protein and albumin, creatinine kinase MB isoenzyme (CK-MB), and C-reactive protein (CRP). Hematology assays included full blood count and white cell differential count and clotting assessed as international normalized ratio (INR). Urinalysis, including dipstick (pH, specific gravity, glucose, protein, ketones, blood) and microscopic examination (sediment, RBCs, WBCs, casts, crystals, epithelial cells, bacteria), was performed.

Fasting blood glucose was measured by the ‘finger prick’ test.

Urinary hydroxyproline output was measured in a 24-h urine sample and was compared at baseline and every 12 weeks thereafter to assess any changes in bone loss after exposure to IV HP-β-CD for up to 48 weeks. An LC-MS/MS bioanalytical method validation was performed by MBL for the quantification of trans-4-hydroxy-L-proline (hydroxyproline) in human urine [42].

AEs and concomitant medications were recorded regularly throughout the study.

As hearing loss is associated with NPC natural disease progression, it is one of the 17 domains measured in the NPC Clinical Severity Score (17D-NPC-CSS), which attributes a score of 0–5 based on the pure tone average across all measured frequencies. Hearing was scored in the patients using this system. Patients underwent audiology testing across a range of frequencies from 0.5 to 8 kHz at screening, baseline and at weeks 12, 24, 36, and 48 using either pure tone audiometry or ABR if the patient was unable to offer behavioral responses to sound stimulation. However, it should be noted that grade shifts in hearing may not result in a significant change in the pure tone average to change the clinical severity score.

### 2.6 Statistical analysis

All analyses in this study were descriptive in nature. Summary statistics included N, mean, median, standard deviation, minimum and maximum for continuous data, and count and percentage for categorical data. Geometric mean was calculated for data from log-normal distributions. The study populations were as follows: Safety - all randomized patients who received at least one dose; Intent-to-treat (ITT) - all patients assigned to treatment regimen even if not dosed; PK - all randomized patients who received a full dose, and pharmacodynamic (PD) - all randomized who received a full or partial dose.

## 3 Results

### 3.1 Patient demographics

Patient enrollment began on 20 June 2017 and the last visit of the last patient was completed on 03 March 2021. A total of 13 patients signed informed consent, one of whom was a screening failure, and 12 patients were enrolled, randomized, and received treatment. Nine of the 12 patients completed the study (2 of 5 in the 1500 mg/kg group; all 4 in the 2000 mg/kg group; all 3 in the 2500 mg/kg group). Three patients (all in the 1500 mg/kg group) discontinued the study, none of them for safety reasons. The reason for discontinuation was physician decision/site PI discretion for 1 patient, withdrawal by subject/patient/parent/guardian for 1 patient, and inability to reach the study site due to COVID travel restrictions for 1 patient. Patient demographic characteristics, baseline disease, 17D-NPC-CSS, and baseline organomegaly are summarized in Table 1.

**Table 1:** Demographic and disease characteristics at baseline.

		HP-β-CD 1500 mg/kg (*N* = 5)‡	HP-β-CD 2000 mg/kg (*N* = 4)§	HP-β-CD 2500 mg/kg (*N* = 3)§	Total (*N* = 12)Φ
Age (years)	Mean	12.2	13.5	10.7	
	(range)	(2, 34)	(2, 39)	(3,21)	12.3 (2, 39)
Sex	Male n (%)	2 (40.0)	3 (75.0)	2 (66.7)	7 (58.3)
	Female n (%)	3 (60.0)	1 (25.0)	1 (33.3)	5 (41.7)
Race⁎	White n (%)	4 (80.0)	4 (100)	3 (100)	11 (91.7)
	Black/African n (%)	1 (20.0)	0	0	1 (8.3)
17D-NPC-CSS score	Mean (SD)	21.0 (9.25)	15.5 (7.42)	17.7 (5.69)	18.3 (7.63)
Hepato-splenomegaly†	Liver and spleen (n)	1	2	1	4
	Liver only (n)	0	0	0	0
	Spleen only (n)	3	2	1	6
	Normal (n)	1	0	1	2

⁎ No other races were represented.

† Abdominal ultrasound at Screening as read by central radiologist (see Methods); numbers represent the number of patients.

‡ All 5 patients in the 1500 mg/kg were receiving miglustat.

§ 3/4 patients in the 2000 mg/kg group and 2/3 patients in the 2500 mg/kg group were receiving miglustat.

Φ All 12 patients in the study were reported to be not Hispanic or Latino.

Demographic characteristics were generally comparable across the three treatment groups. The 17D-NPC-CSS score was slightly higher in the 1500 mg/kg cohort compared with in the 2000 and 2500 mg/kg cohorts.

### 3.2 Pharmacokinetics

**Fig. 1:**
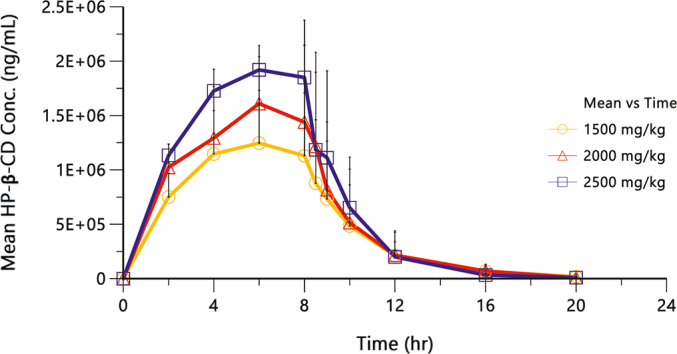
Mean (SD) Plasma Concentrations (ng/mL) of HP-β-CD versus Time in Patients Following an Initial IV Infusion of HP-β-CD Over 8 Hours at 1500, 2000, or 2500 mg/kg.

Mean plasma HP-β-CD concentrations over time are shown in Fig. 1.

A summary of mean HP-β-CD plasma PK parameters derived using non-compartmental methods (i.e., the model does not rely on assumptions about body compartments) is shown in Table 2.

**Table 2:** Summary of mean pharmacokinetic parameters for HP-β-CD in plasma from 11† patients after an initial IV infusion of HP-β-CD at 1500, 2000, or 2500 mg/kg.

	t_1/2_	t_max_⁎	C_max_	AUC_0-∞_	CL	Vd
	(hr)	(hr)	(ng/mL)	(hr*ng/mL)	(mL/h/kg)	(mL/kg)
HP-β-CD, 1500 mg/kg, IV infusion						
n	5	5	5	5	5	5
Mean	2.01	6.00	1,272,600	11,604,437	152	426
SD	0.265	(3.98–8.00)	489,692	4,718,654	72.7	168
HP-β-CD, 2000 mg/kg, IV infusion						
n	3	3	3	3	3	3
Mean	1.63	6.15	1,856,667	13,805,001	172	399
SD	0.149	(5.92–8.03)	803,140	7,029,477	83.8	193
HP-β-CD, 2500 mg/kg, IV infusion						
n	3	3	3	3	3	3
Mean	1.81	6.00	1,920,000	16,133,582	156	412
SD	0.259	(6.00–6.05)	121,655	1,814,458	17.9	107

⁎ Median (min-max) was used for t_max_.

† Only 11 of 12 patients are included in the table. For Patient 11 in the 2000 mg/kg group, samples were collected on Day 1 only, so this patient is not included in the table.

#### 3.2.1 Pharmacokinetics of HP-β-CD in plasma

Following IV administration of HP-β-CD in patients, plasma t_max_ was calculated to be 6 h (median t_max_) for the 1500 mg/kg and 2500 mg/kg groups and 6.15 h for the 2000 mg/kg group. Plasma exposure (AUC_0-∞_) and C_max_ increased in a slightly less than dose-proportional manner (Fig. 1 and Table 2). Upon a 1.7-fold increase in dose from 1500 to 2500 mg/kg of HP-β-CD, the mean plasma AUC_0-∞_ increased by 1.4-fold, and the mean plasma C_max_ increased by 1.5-fold, see Table 2.

It should be noted that small numbers of patients in those groups resulted in large variations in standard deviation (SD) in AUC_0-∞_.

#### 3.2.2 HP-β-CD concentrations in CSF

Trappsol**®** Cyclo™ was detectable in CSF at the first timepoint evaluated, 4 h post-start of infusion, and levels remained at maximum or close to maximum at the last timepoint evaluated, 12–13 h post-start of infusion (4–5 h following the end of infusion). For the 5 individuals for whom all four data points were available (0, 4, 8, 12 h), CSF levels of the drug were detected 4 h after the end of IV infusion. In one patient with a single assessment point (at 8 h from the start of infusion), HP-β-CD was not detected.

As anticipated, variability in concentrations of HP-β-CD in CSF post-infusion was observed in the 7 patients who had more than one data point across the three groups (1 patient had only one data point). Five patients were in the range 11.5 to 35.3 μM at peak level; however, 2 patients had much higher peak values of 241.1 and 303.4 μM, respectively, see Table 3.

**Table 3:** HP-β-CD in CSF.

Patient ID	Hr after SOI	Plasma conc (ng/mL)	CSF conc (ng/mL)	CSF conc (μM)	Dose (mg/kg)	Age (Y)	NPCSS Total at Baseline
1	Predose	0	BLQ	BLQ	1500	34	29
	4	1,470,000	6890	4.8			
	8	1,930,000	22,200	15.2			
	12	573,000	36,700	25.0			
2	Predose	0	BLQ	BLQ	2500	21	24
	4	1,770,000	307,000	210.3			
	8	2,060,000	352,000	241.0			
	12	36,000	147,000	100.7			
3	Predose	0	BLQ	BLQ	2000	39	12
	4	1,970,000	7510	5.1			
	8	2,780,000	20,900	14.3			
	12	546,000	24,900	17.0			
4	Predose	0	BLQ	BLQ	1500	15	21
	4	1,590,000	199,000	136.3			
	8	1,370,000	443,000	303.4			
	12	184,000	179,000	122.6			
6	Predose	0	BLQ	BLQ	2000	11	26
	4	1,440,000	13,100	9.0			
	8	1,260,000	48,800	33.4			
	12	68,300	24,900	17.0			
8	Predose	0	BLQ	BLQ	2500	3	16
	4	1,700,000	51,600	35.3			
	8	ND	ND				
	12	ND	ND				
11	Predose	0	BLQ	BLQ	2000	2	9
	4	ND	ND				
	8	599,000	BLQ	BLQ			
	12	ND	ND				
12	Predose	ND	ND		2500	11	13
	4	ND	ND				
	8	ND	16,800	11.5			
	12	ND	ND				

BLQ – below the limit of quantification; ND – not determined; SOI – start of infusion.

Patients 5,7 and 10 had no post-infusion samples.

Individual patient panels showing maximum percentage change from baseline in cholesterol biomarkers, maximum concentration of HP-β-CD in CSF and clinical parameters at baseline and weeks 12, 24, 36 and 48 are included in Appendix A1.

### 3.3 Pharmacodynamics

Target engagement was shown by direct effects on central and systemic biomarkers of cholesterol synthesis, metabolism, and catabolism. Four patients had complete PD data sets, as presented below. Data from the remaining patients are not presented due to the inability to collect samples for PD assessments (several missing samples because of age-related blood volume restrictions and missed visits due to the COVID-19 pandemic).

**Fig. 2:**
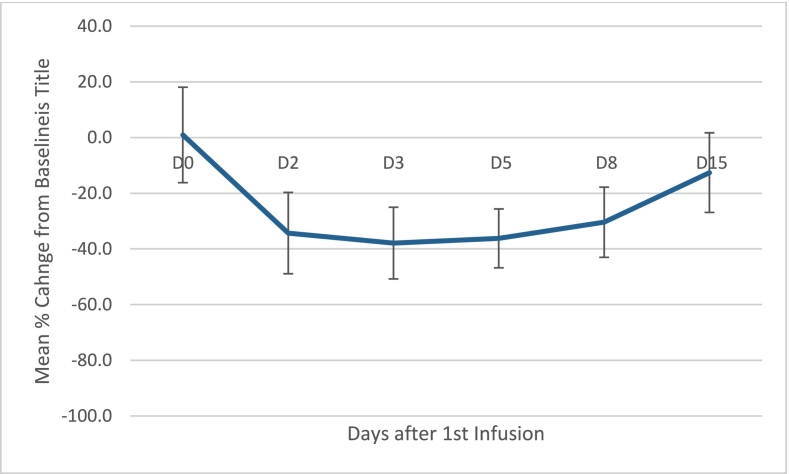
Mean % change from baseline in serum lathosterol after 1st infusion (*n* = 4). Figure based on 4 patients with a complete data set. Bars represent standard error of the mean.

Serum lathosterol concentration decreased rapidly within 24 h following the initial infusion, resulting in nearly 40% (37.9%) reduction of the baseline concentration 3 days after the initial infusion of HP-β-CD, see Fig. 2. The onset of treatment effect was rapid (within 24 h of the first infusion) and clinically relevant.

Across the dose range, the greatest reduction in lanosterol (another precursor) from baseline was at Day 3 (−28.12%; *n* = 6; data not shown). Desmosterol generally showed little change over the course of the study (data not shown).

#### 3.3.1 Cholesterol metabolites

**Fig. 3:**
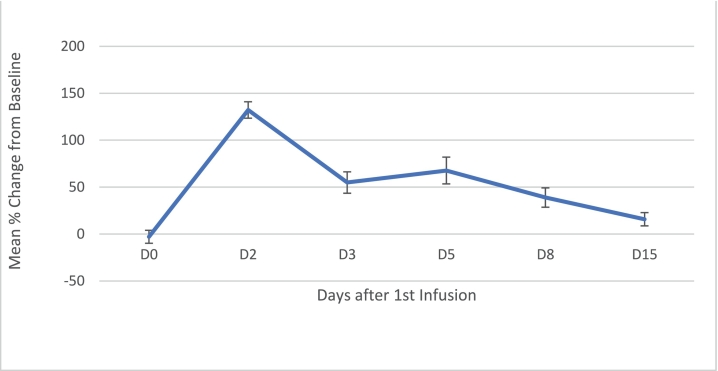
Mean % change from baseline in serum 4β-hydroxycholesterol after 1st infusion (n = 4). Figure based on 4 patients with a complete data set. Bars represent standard error of the mean.

The serum concentration of 4β-hydroxycholesterol peaked at 132.2% of baseline on Day 2 after the initial infusion, see Fig. 3.

4β-Hydroxycholesterol is one of the major oxysterols in humans. Excess cholesterol released by HP-β-CD is converted to oxysterols, which is why oxysterol levels rise shortly after Trappsol**®** Cyclo™ infusion. These oxysterols eventually get converted to bile acids and secreted, which is one of the ways the body gets rid of excess cholesterol. As the sequestered cholesterol pool becomes depleted with each subsequent Trappsol**®** Cyclo™ infusion, the rise in oxysterols post-infusion becomes smaller and smaller.

**Fig. 4:**
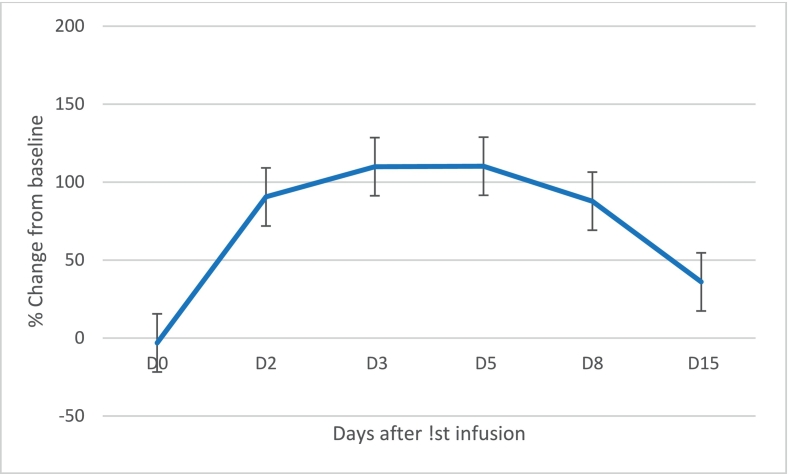
Mean % change from baseline in Serum 27-hydroxycholesterol after 1st infusion (*n* = 4). Figure based on 4 patients with a complete data set. Bars represent standard error of the mean.

27-Hydroxycholesterol, another metabolite of cholesterol, peaked with an increase of 110.2% above baseline on Day 5 (see Fig. 4) and 25-hydroxycholesterol had a smaller rise of 45.3% (*n* = 6) above baseline at Day 5 after the initial infusion (data not shown). Except for the first dosing and interim collection of samples until the next dosing, there was no intention to measure these metabolites at other intervals or to conduct inter-dose analyses.

**Fig. 5:**
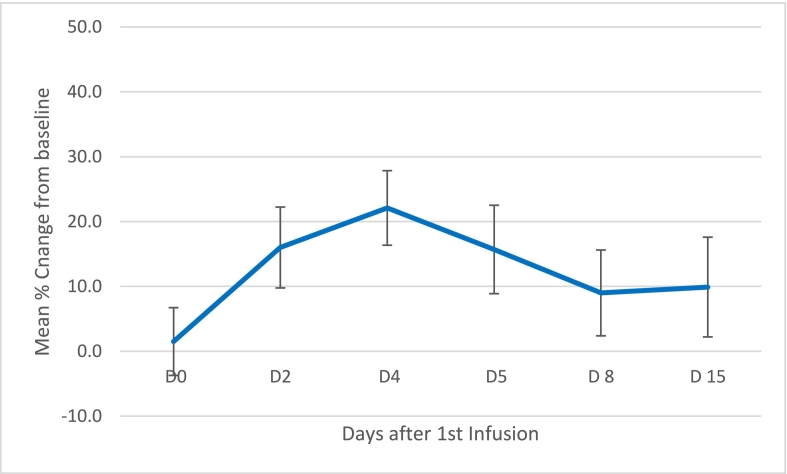
Serum 24S-hydroxycholesterol after 1st infusion. Figure based on 4 patients with a complete data set. Bars represent standard error of the mean.

The serum level of 24S-hydroxycholesterol increased to a peak of 22% above baseline on Day 4 after the first infusion (taken as a mean) (see Fig. 5). At Week 48, the mean level of 24S-hydroxycholesterol was roughly 6% lower than the mean level at baseline, possibly indicating a slight overall reduction in CNS cholesterol (data not shown).

#### 3.3.2 Disease-related biomarkers

##### 3.3.2.1 Plasma and PBMC biomarkers

**Fig. 6:**
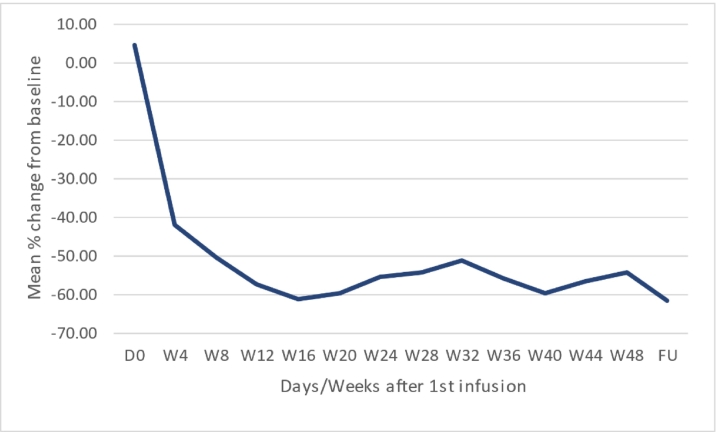
Mean % change from baseline in plasma PPCS. Figure based on 4 patients with a complete data set.

The plasma level of PPCS decreased to a low of −61.3% of baseline at Week 16 (see Fig. 6), and this level was maintained during the treatment period and at the follow-up visit, 28 days after the last treatment.

**Fig. 7:**
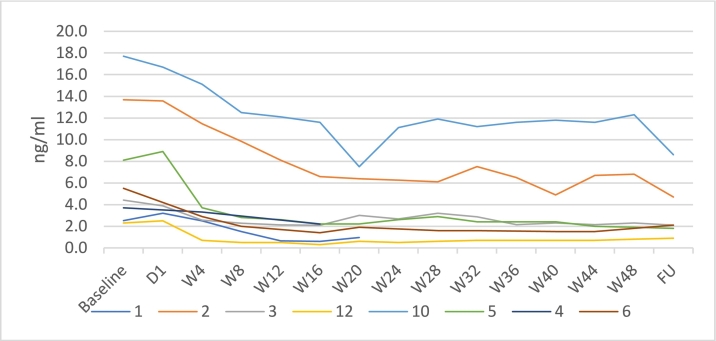
Plasma PPCS levels. Patients 7, 8, 9 and 11 were missing because of age-related blood volume restrictions or missed visits due to the COVID-19 pandemic.

Individual plasma PPCS levels are shown in Fig. 7. Each patient has a different color that corresponds to their patient number, as shown in the legend.

The data show a variable decline in PPCS levels across individual patients. The rapid reduction in PPCS levels reached a peak 3–5 days after the start of the initial infusion and levels remained well below baseline until the end of the study.

Due to the strict and narrow timetables involved in maintaining sample viability for Lysotracker PBMC samples, coupled with the caps on maximum blood samples allowed in young patients, the numbers of viable samples tested were fewer than expected. For available samples, no trends in size of relative acidic compartment, as measured by Lysotracker, were observed either on an individual basis or when taken as a group (data not shown).

##### 3.3.2.2 CSF biomarkers

**Fig. 8:**
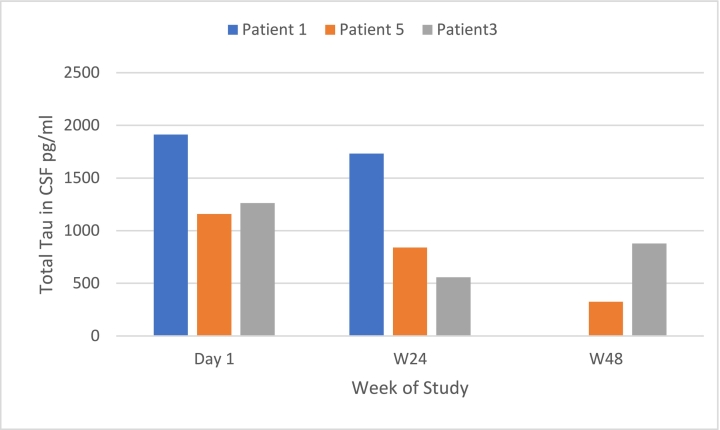
CSF total tau (pg/mL). Patient 1, aged 34 years, NPCSS at baseline was 16; Patient 5, aged 24 years, NPCSS at screening was 24; Patient 3, aged 39 years, NPCSS at baseline was 12. Patient 3 sample collected at Week 26 not Week 24.

All 3 adult patients who participated in the trial underwent optional lumbar puncture. In the 3 patients for whom serial samples were collected, reduction in total Tau levels from baseline was observed at 24, 26 or 48 weeks, see Fig. 8. High baseline levels of total Tau can be attributed to breakdown of neurons releasing this protein, and elevated levels may be present in patients with NPC [43].

The other potential biomarkers measured in the CSF in this study, GFAP and TNFα, yielded little or no information because nearly all the levels were below the lower limit of quantification (LLQ) of 12.5 pg/mL for Tau, 156 pg/mL for TNFα and 156 pg/mL for GFAP.

### 3.4 Efficacy outcomes

#### 3.4.1 Morphology of liver and spleen

Importantly, no patients experienced a worsening in hepatomegaly and/or splenomegaly relative to baseline but showed improvement or were unchanged. Liver evaluations were available at screening and 48 weeks in 6 patients. No patients experienced worsening and 2 patients (both aged 2 years) showed signs of improvement.

Nine patients had spleen evaluations at Screening and Week 48, 7 showed no change, 2 patients improved/normalized.

No negative changes in hepatic safety laboratory parameters (i.e., aspartate aminotransferase [AST], alanine transaminase [ALT], total bilirubin, and direct bilirubin) were observed. Platelet levels remained stable.

A qualitative assessment of liver and spleen size from Screening to Week 48 is shown in Appendix A2 and A3, respectively.

#### 3.4.2 Assessment of clinical severity

Changes in 17D-NPC-CSS ratings for patients who completed the study are presented in Table 4.

**Table 4:** 17D-NPC-CSS domains showing improvement or worsening at end of study.

Patient Number (age, years)	Improvement in Individual Domains at Week 48 compared with baseline	Worsening in Individual Domains at Week 48 compared with baseline	NPCSS Total at baseline	NPCSS Total at Week 48 or end of study
2 (21)	**Swallow** **− 1** Seizures −2 Gelastic Cataplexy −1 Incontinence −1	**Fine Motor Skills +** **1** **Cognition +** **1**	**24**	**21**
3 (39)	Eye Movement −1 **Fine Motor Skills** **− 1** Psychiatric −1	None	12	9
5 (4)	Gelastic Cataplexy-1 Auditory brainstem response −1 Memory −1	None	24	21
6 (11)	**Ambulation** **− 1** **Swallow** **− 2** Gelastic Cataplexy −2 Hyperreflexia −1 Narcolepsy −1 Incontinence-1 Behavior −1	**Speech +** **1** **Fine Motor Skills +** **2** Hearing +2	**26**	**22**
7 (2)	**Ambulation** **− 3** **Fine Motor Skills** **− 1**	**Speech +** **1** Hyperreflexia +1	**5**	**3**
8 (3)	Eye Movement −1 **Speech-1**	**Fine Motor Skills +** **1** Hearing +2	**16**	**17**
10 (2)	None	**Cognition +** **2** **Fine Motor Skills +** **1** Hyperreflexia +1	**15**	**19**
11 (2)	Eye Movement −1 **Cognition** **− 2**	**Fine Motor Skills +** **1** Memory +1	**9**	**8**
12 (8)	Gelastic Cataplexy −1 Incontinence −1	**Ambulation +** **1** **Fine Motor Skills +** **1** Memory +2 Hyperreflexia +1	**13**	**16**

Patient 1 withdrawn at Week 24, Patient 4 no consent for 17D-NPC-CSS, Patient 9 unable to attend Week 48 due to COVID-19.

Domains in **bold** text are those determined by NPC families and their caregivers, in collaboration with the FDA [44], to be the most important for quality of life.

Nine of 12 patients completed the assessment for clinical disease severity in this 48-week study. The primary efficacy outcome measure for this study was at least a 1-point reduction (improvement) in 2 or more of the 17D-NPC-CSS domains. Eight of the 9 patients (89%) who completed the study met this endpoint. Of these 8 patients, 6 (75%) improved in at least 1 of the 5 domains judged by patients and their caregivers to be the most important for their quality of life [44]. One patient worsened overall using the 17D-NPC-CSS measure.

One potential additional clinical benefit with derived effects on quality of life was observed with respect to incontinence. Four of nine patients who completed the study showed improvement in this disease feature: data for three patients are shown in Table 4, and data for one patient (a 2-year-old) are not shown. The site PI did not score the Incontinence domain for the 2-year-old patient due to practical challenges. One of these patients (patient 6) became toilet trained during the course of the study, which was an unexpected finding.

#### 3.4.3 Clinical global impression

Using the Clinical Global Impression of Improvement (CGI—I) Scale, 7 of the 9 patients who completed the study were viewed by their treating physicians as having improved (1 very much; 1 much; 5 minimally) at the end of the study compared with baseline, and 2 were viewed as unchanged (remained stable) (see Appendix A4). Improvement or stabilization is considered a successful outcome in a progressive disease.

Using the PGIC scale, all 9 patients who completed the study experienced improvement or showed no or little change (remained stable) at Week 48 (see Appendix A4).

#### 3.4.4 Pediatric quality of life outcomes

The low numbers of patients in each dose group for pediatric quality of life outcomes using the Pediatric Quality of Life survey meant it was difficult to provide a meaningful interpretation of averaged data.

#### 3.4.5 Additional clinical assessments of ataxia

The SARA, an 8-item performance-based scale of cerebellar ataxia (0, no ataxia to 40, most severe ataxia), is a widely used clinical outcome measure. In the present study, SARA was measured every 12 weeks.

**Fig. 9:**
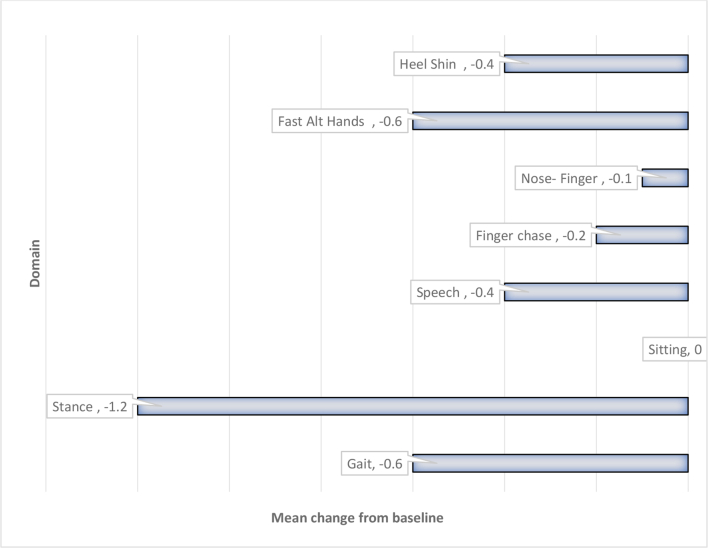
Change from baseline in mean SARA scores at week 48.

Ataxia outcome assessments, as measured using the SARA, are shown in Fig. 9.

Results from the SARA show an improvement in the mean score in 7 of 8 domains at Week 48 compared with baseline, with the most notable improvements in the domains of stance, gait, and fast alternating hand movements. Importantly, there was no overall worsening in any domain.

There was no change in the mean score for 1 of the SARA domains, sitting: all patients had a score of 0 (normal) or 1 (slight difficulties) at baseline, leaving little room for improvement, and these scores were not changed in any individual patient at the 48-week timepoint. As expected, there was no correlation to dose, given the small number of patients per study cohort.

Bead-threading was used as another supplementary assessment of fine motor skills. The age of study participants precluded analysis of a robust data set. Two patients showed marked improvement in ability to place beads on a thread in the span of 1 min.

### 3.5 HP-β-CD observed safety and tolerability profile

#### 3.5.1 Adverse events

Trappsol**®** Cyclo™, administered intravenously every 2 weeks over the course of 48 weeks, was well tolerated overall in pediatric and adult patients with NPC, irrespective of baseline disease severity. All three dose levels of HP-β-CD showed an acceptable safety and tolerability profile, with no clinically significant events, changes, or trends considered related to study treatment noted across safety laboratory parameters, physical examinations, vital signs, or electrocardiograms. A total of 185 treatment-emergent adverse events (TEAEs) were reported across the three cohorts (Table 5). No deaths were reported and no patients were withdrawn or withdrew due to a TEAE. Fifteen serious adverse events (SAEs) were reported, with 14 considered to be treatment-emergent.

**Table 5:** Summary of adverse events by treatment.

Parameter	1500 mg/kg (*N* = 5) n (%)	2000 mg/kg (*N* = 4) n (%)	2500 mg/kg (*N* = 3) n (%)	Total (*N* = 12) n (%)
Total Number of AEs	64	66	74	204
Total Number of TEAEs	51	62	72	185
Total Number of Serious AEs	9	2	4	15
Total Number of Serious TEAEs	8	2	4	14
Number of Patients with at Least One TEAE	5 (100)	4 (100)	3 (100)	12 (100)
Number of Patients with at Least One Related TEAE⁎	3 (60.0)	2 (50.0)	2 (66.7)	7 (58.3)
Number of Patients with at Least One Severe (Grade ≥ 3) TEAE†	2 (40.0)	1 (25.0)	2 (66.7)	5 (41.7)
Number of Patients with at Least One TEAE Leading to Death	0	0	0	0
Number of Patients with at Least One Serious TEAE	2 (40.0)	1 (25.0)	2 (66.7)	5 (41.7)

AE, adverse event; TEAE, treatment-emergent adverse event.

⁎ TEAE with missing relationship was counted as related. If a patient had multiple TEAEs with different relationships to study drug, only the related event was summarized in the table.

† Missing severity counted as severe.

At total of 2/5 patients in the 1500 mg/kg group, 1/4 patients in the 2000 mg/kg group, and 2/3 patients in the 2500 mg/kg group experienced at least one severe (Grade ≥ 3) TEAE.

The most common TEAEs as a percentage of the total number of infusions were seizure (18.3%), rash (14.3%), rhinitis (12.1%) and cataplexy (11.4%) (see Appendix A5), with all other TEAEs observed in <10% of patients. The majority of TEAEs were considered by the site PI to be not related to study drug but rather associated with NPC and its complications, or the illnesses associated with a young population. All patients who experienced AEs of seizures or cataplexy had a medical history of seizure and/or cataplexy.

Most of the SAEs were considered unrelated. Two SAEs were considered by the site PI to be related to treatment. One event was change in hearing that did not meet formal ototoxicity criteria, and one event was peripheral/hand swelling and erythema around the cannula site. These events were considered to be mild but related to study treatment; both events resolved and the patients completed the study.

Three patients each experienced one event that required hospitalization; none of the events were considered by the site PI to be related to the study treatment. One event (1500 mg/kg group) was a CTCAE grade 4 TEAE of aspiration pneumonia, one event (1500 mg/kg group) was intermittent hospitalization due to seizures and poor compliance with antiepileptic therapy, and one event (2000 mg/kg group) was due to complications following a lumbar puncture (CSF leak) performed as part of the study assessments.

The independent safety review committee performed periodic review of safety data and found the benefit-risk profile appropriate based on the safety and tolerability profile of the three doses of HP-β-CD in this study.

#### 3.5.2 Hearing

No safety signals or trends were noted apart from audiometry changes with no perceptible changes in hearing. Slight-to-mild hearing loss in two cases and transient hearing change in three cases were reviewed by the SRC and a retrospective analysis performed by an experienced practicing clinical audiologist consultant.

Hearing was measured by behavioral testing in all but one patient, who could not comply and had ABR testing. Of the changes in hearing noted below, none were reported by either the patient or caregiver as a noticeable change and no other factors that would have accounted for the change could be ascertained. In addition, upon review of individual PK data (e.g., AUC), no apparent association between the level of systemic drug concentrations and patients who experienced a TEAE related to hearing functionality was identified.

In the 1500 mg/kg treatment group (N = 5), 3 patients (aged 4–34 years) showed no worsening or change from baseline through to their end of study hearing assessment. Of the 2 patients who showed a change from baseline, patient 7 (young pediatric), with normal baseline hearing, experienced a temporary threshold shift in hearing mid-study, returning to the baseline values by the end of the study, indicating no permanent change to the patient's hearing. The other, patient 9, experienced a worsening of hearing at Week 12 and this remained at this level throughout the remainder of the study with no further deterioration.

In the 2000 mg/kg treatment group (*N* = 4), 2 young pediatric patients (aged 2 years) showed no change from their initial baseline hearing level. Of the other 2 patients who showed a temporary change from their baseline hearing, patient 3 (adult) had normal hearing at baseline but experienced a temporary threshold shift in hearing mid-study, which returned to baseline values upon study completion with no permanent change. Of note, the deterioration was only reported at frequencies evaluated outside those required by the protocol; therefore, interpretation of this deterioration with respect to the natural history of hearing in NPC patients is unknown. Patient 6 (older pediatric) with normal hearing at baseline, experienced a deterioration in their hearing at the Week 48 visit; hearing assessment 4 weeks after study completion showed that the hearing thresholds had returned to their baseline.

In the 2500 mg/kg treatment group (*N* = 3), patient 2 (young adult) and patient 12 (pediatric) showed no change from a normal baseline until their end of treatment hearing assessment. The final patient in this treatment group, patient 8 (young pediatric) had a normal hearing at baseline and showed a slight-to-mild deterioration at the Week 24 audiology assessment. This deterioration remained at this level throughout the rest of their participation on the study but did not worsen.

The hearing (17D-NPC-CSS) grade shift data for each patient in the study are shown in Table 6.

**Table 6:** Hearing (17D-NPC-CSS) grade shift data for each patient in the study.

		As assessed by 17D-NPC-CSS Score taken from the performed PTA or ABR,⁎ where applicable		
Treatment Group	Patient Number	Baseline Hearing Score†	End of Treatment (EOT) Score‡	Patient Notes
1500 mg/kg				
N = 5	1	3	3	Week 24 (EOT due to patient withdrawal)
	4	0	ND	Withdrawal after week 12 but no reported change by patient or family throughout
	5	ABR = 1	ABR = 0	No worsening of hearing compared with baseline.
	7	0	0	
	9	0	2	A deterioration but stable and not noticed by family
2000 mg/kg				
N = 4	3	0	0	Scored 2 mid-study but returned to normal
	6	0	2	Additional audiology assessment performed 4 weeks after study completion and was reported as normal
	10	ABR = 0	ABR = 0	
	11	2	2	
2500 mg/kg				
N = 3	2	0	0	
	8	0	2	A deterioration but stable and not noticed by family
	12	ABR = 0	ABR = 0	

ABR – auditory brainstem response; 17D-NPC-CSS – 17-Domain Niemann-Pick disease Type C-Clinical Severity Scale; ND – not determined; PTA – pure tone average.

17D-NPC-CSS Hearing Loss Grading. 1 = High frequency loss. 2 = Slight to mild. 3 = Moderate. 4 = Severe. 5 = profound.

⁎ ABR scoring. 0 = Normal. 1 = Abnormal.

† Baseline is the last hearing assessment performed before the first dose is administered, i.e., at screening or the baseline visit.

‡ In the event of an early withdrawal, end of treatment is the final hearing assessment performed.

### 3.6 Descriptive review of patient profiles

Appendix A1 contains patient profiles with highlights of PK, PD, and efficacy outcome measures. Four of 12 patients had a complete time course for both clinical endpoint data sets and relevant biomarkers. Three of these 4 patients (75%) showed improvement, as measured by either or both of the 17D-NPC-CSS or CGI-I scales, and had clinically relevant changes in disease-related biomarker (PPCS) or biomarkers of target engagement (24S-hydroxycholesterol and lathosterol). Patient 12 worsened on the 17D-NPC-CSS but remained stable on the CGI—I: this patient's biomarkers trended in the direction of benefit.

## 4 Discussion

The primary and secondary outcome measures of this 48-week study demonstrated systemic and central exposure of Trappsol**®** Cyclo™ following IV administration, confirming Phase I results [45] with effects on cholesterol metabolism and catabolism. Importantly, the 48-week treatment period allowed evaluation and demonstration of clinical benefits. All analyses in this study were descriptive in nature; no formal statistical analysis was performed. In this study, all three doses of IV Trappsol**®** Cyclo™ improved key clinical signs and symptoms, and were associated with stabilization or improvement in overall disease severity. Overall, there were no unexpected TEAEs observed and Trappsol**®** Cyclo™ was generally well tolerated in both pediatric and adult patients, with comparable safety profiles in this 48-week study and that reported in the 12-week Phase I study [45]. Further, the findings of the present study are consistent with previously published findings from several individual compassionate use programs [46].

An early clinical approach, by a different group, to the treatment of the neurological symptoms of NPC1 with another HP-β-CD was to administer the drug via the intrathecal (IT) route [47,48] because it was assumed that this molecule would not cross the blood-brain barrier. High concentrations of HP-β-CD in the brain resulted in permanent hearing loss due to damage to hair cells in the cochlea [49]. Due to a combination of the problems with ototoxicity, the risk of infection, and the highly invasive nature of the IT route, coupled with the favorable safety and efficacy signals observed with IV treatment in compassionate use programs, the IV route was chosen to be investigated in the current study [46].

In this study, Trappsol**®** Cyclo™ was administered intravenously to reach both central and peripheral compartments. In theory, the IV route offers several potential advantages, such as treatment of peripheral organs, a more balanced PK profile of Trappsol**®** Cyclo™ reflected in a favorable benefit-risk profile, and clear indication that therapeutic concentrations must be present in the CNS compartment, given the observed effects on neurological signs and symptoms.

17D-NPC-CSS is a disease-specific instrument designed to quantify change in a wide range of NPC1 symptoms [35]. All 17 domains were evaluated in the present study to gain as much information as possible on disease manifestation in each patient. Using a protocol-defined efficacy success criterion of improvement in at least 2 of the 17 domains of the scale, 89% of patients met the standard in the current study.

A relevant paradigm shift in assessment of efficacy enabled focus on 5 major and highly relevant domains (fine motor skills, swallow, ambulation, cognition, and speech), which are deemed the most important by treating physicians, carers, and the major health authorities [44]. Using the same efficacy criterion based on the 5-domain scale, 75% of patients still met the efficacy criterion. Overall, improvement or stabilization was seen in the majority of patients. A small number of patients worsened, which may be attributed to overall disease-related decline or patients having disease too advanced to experience full clinical benefits.

One of the important outcome measures used in the study was CGI-I [40], which is designed to measure change in overall disease symptoms, as rated by a physician. Improvements using CGI-1 were seen in 7 patients; 78% of those rated at 48 weeks. The patient-reported treatment benefits using the PGIC scale [41] were consistent with the evaluation performed by the site PIs with CGI-I and shows that 48 weeks of treatment results in all patients responding with either improvement or stabilization, which is critical in a progressive neurodegenerative disease. Descriptively, this outcome is superior to that reported for other investigational products, such as arimoclomol, with which only 58.8% patients improved/stabilized at 12 months and CGI-I outcome was indistinguishable from that in patients who received placebo [50]. A limitation of this comparison is that the arimoclomal study was placebo-controlled [50], whereas the current study had no placebo or other control. In addition, the sample sizes of the studies were disparate, with the current study limited to 9 patients who completed the trial, compared to 42 patients for the arimoclomol trial.

Consistent improvement was also observed in the SARA, with improvements in all domains except sitting, as all patients had a score of 0 (normal) or 1 (slight difficulties) at baseline, leaving little room for improvement. These data further support the positive clinical benefits of Trappsol**®** Cyclo™ on multiple aspects of neurological functioning, with demonstrated improvement or stabilization after nearly 1 year of treatment.

Evaluation of peripheral effects included abdominal ultrasound (hepatic and splenic assessments). No clinically relevant effects on hepatomegaly and/or splenomegaly were observed, although there was indication of reduction or stabilization of hepatomegaly and/or splenomegaly. The lack of obvious change in spleen and liver morphology does not rule out improvement in organ function, possibly resulting from correction of cholesterol imbalance.

Results from the current study confirm previous findings [45] that HP-β-CD penetrates the CSF during and after IV infusion. Analysis of drug concentrations in CSF serves as an acceptable surrogate, as direct measurements of HP-β-CD in nervous tissue within the brain compartment is not feasible. Pharmacokinetics of HP-β-CD in the CSF appear to differ from those in plasma as measurable levels are present in CSF when levels in plasma are undetectable. Further investigation of the PK of HP-β-CD in the CSF are needed before making any precise conclusions about the differences observed.

The Phase I study [45] provided direct, unequivocal evidence of the release of cholesterol from liver cells, as demonstrated by significant reduction in/clearance of hepatic lipid content, observed using filipin staining, following only 12 weeks of treatment. Increase in the serum concentration of cholesterol metabolites, oxysterols, or bile acid precursors, indicates an increase in the breakdown of cholesterol [30]; therefore, the cholesterol metabolites 4β-, 24S-, 25-, and 27- hydroxycholesterol were measured in the current study. Although there were no direct measurements of cholesterol release in this study, there was a reduction in cholesterol synthesis precursors (lathosterol and lanosterol) and an increase in cholesterol metabolites (4β-hydroxycholesterol and 27-hydroxycholesterol) over 3–5 days after the first infusion. This indicates release of trapped cholesterol and a possible resetting of cholesterol homeostasis. This matches the pattern seen in the Phase I study [45] and clearly indicates a change in cholesterol homeostasis resulting from a large release of trapped cholesterol into the peripheral circulation with rapid onset of drug effect and durable effect. Cholesterol precursors and metabolites were assessed in the current study as markers of cholesterol release or metabolism; it was not the intention to assess these precursors and metabolites as biomarkers of NPC1. The current study data indicate a trend towards normalization of precursor and metabolite levels, and overall cholesterol metabolism in the patients.

Evidence of the biological activity of HP-β-CD and target engagement is provided by two critical biomarkers: 24S-hydroxycholesterol [51] and total Tau [43]. The first biomarker, 24S-hydroxycholesterol, is a cholesterol metabolite specific to the brain [51]. Cholesterol synthesis and metabolism in the brain is isolated from the peripheral circulation. Any change in cholesterol homeostasis will be reflected by a change in the level of 24S-hydroxycholesterol due to its ability to cross the blood-brain barrier into the peripheral circulation [51]. Shortly after Trappsol**®** Cyclo™ administration, a small increase in 24S-hydroxycholesterol in the serum was observed in both the Phase I study [45] and the current study, and was interpreted as an indicator of release of cholesterol trapped in the cells of the central nervous system, with an ensuing change in cholesterol turnover to prevent problems due to excess cholesterol.

The second biomarker, Tau, a microtubule-associated protein, is a marker of neuronal loss and neurodegeneration [43]. Levels of total Tau in CSF in healthy individuals are generally 200–300 pg/mL [52]; for example, Okamura reported a mean (standard deviation) of 230.1 (92.4) pg/mL in a normal population [53]. There was a trend towards a reduction in total Tau following treatment with HP-β-CD in the current study. These data were comparable to those in the Phase I study [45]. Both biomarkers provide evidence that HP-β-CD releases cholesterol from cells in the brain and may provide a metabolic milieu where the rate of neuronal apoptosis is reduced, and cells have an opportunity to restore normal functioning.

PPCS plasma concentrations have been shown to be significantly elevated in NPC1 subjects (mean 2492 ng/mL; range 254–18,200 ng/mL; healthy controls <2.5 ng/mL) [54,56]. PPCS is increasingly used as a diagnostic biomarker for NPC [[54], [55], [56]] and may emerge as a potential biomarker for NPC clinical progression [54]. The precise way in which it links to treatment benefit, irrespective of the mechanism of action of the investigational product, remains unclear. The data show a decline in PPCS levels across individual patients, with baseline levels similar to those of Giese et al. [54]. It was not possible to draw conclusions about levels of PPCS and dose in relation to demographic features of the population in the current study.

The data from the study were limited by the small sample size and, in some cases, infrequent sampling and missing data as a result of the impact of the COVID-19 pandemic. The majority of patients (10 of 12 patients), entered the study on stable doses of miglustat. The use of miglustat is not considered to have impacted the ability to effectively assess the clinical benefits of Trappsol**®** Cyclo™, as patients entered on stable doses of miglustat and therefore had achieved any clinical benefits expected from this drug. Also, by meeting eligibility criteria, the patients were still not effectively treated for their neurological clinical signs and symptoms. Furthermore, no clinically meaningful effect of miglustat treatment on visceral/systemic clinical signs or symptoms has been reported. The clinical treatment benefits and effects on relevant disease-related biomarkers following 48 weeks of treatment with Trappsol**®** Cyclo™ were evident. Finally, this study did not include a control group. Given the length of the study, patients were considered to be their own controls. This design may confound interpretation of the data.

Significant and, for some patients, irrevocable reduction of hearing abilities has been observed following IT administration of other cyclodextrin formulations [48]. The observed effects on hearing following treatment with IV administration of Trappsol® Cyclo™ in the Phase I study [45] and the current study were manageable, with no patients experiencing complete loss of hearing nor requiring hearing aids as a consequence of treatment, and with an appropriate benefit-risk profile. Given that no hearing change was noted by patients or carers, the benefit/risk of continuing treatment with HP-β-CD was considered to be acceptable.

Intravenous administration of Trappsol**®** Cyclo™ was well tolerated, with an appropriate safety profile and no new safety signals, and was considered appropriate across the age and disease spectrum in patients with NPC disease. There was no evidence of any untoward effects of Trappsol**®** Cyclo™ on core organ systems (cardiovascular, respiratory, renal, hepatic, gastrointestinal or CNS). No clinically significant effects or trends were observed for any of the clinical safety laboratory parameters. In addition, no negative effects or trends were observed for vital signs, ECG, physical examination, or abdominal ultrasound (liver, spleen).

This 48-week trial is the longest to date to evaluate the safety, tolerability, and efficacy of IV administration of HP-β-CD in NPC1 patients. Relative reduction in disease progression was observed in 67% of patients who completed the treatment period, as shown by improvement or stabilization in the total score of 17D-NPC-CSS and the 5D-NPC-CSS scales. The results of this study support that Trappsol**®** Cyclo™, by its mechanism of action, has the potential to address the primary etiology of NPC, namely the disrupted cholesterol and lipid pathways, known as the main culprit in NPC. The critical preclinical data [28,29,32]. translated into clinically relevant results in humans, as shown by both the PD data and the clinical benefits observed in both pediatric and adult patients with NPC1. These data reinforce the therapeutic potential of HP-β-CD for addressing the systemic and CNS manifestations of NPC1 and support the drug to become the first targeted therapy for this devasting neurovisceral degenerative disease. Combined with an acceptable risk-benefit profile, these data enabled the design of the global Phase III pivotal efficacy trial now underway to evaluate Trappsol**®** Cyclo™ as a disease-modifying treatment in this patient population.

## 5 Conclusions

Trappsol**®** Cyclo™, administered intravenously every 2 weeks, represents a pharmacological option to effectively improve the pathologic retainment of cholesterol in the lysosomal compartment, restoring the disrupted cholesterol metabolism, and establishing a new equilibrium with derived clinical benefits in patients with NPC. Trappsol**®** Cyclo™ IV has the potential to improve the standard of care in patients with NPC and provide neurological and systemic treatment benefits in this severely impacted patient population with high unmet medical needs.

## Funding

This study was funded by Cyclo Therapeutics, Inc., Gainesville, FL, USA.

## Declaration of Competing Interest

None.

## Figures and Tables

**Table:** 

Pt 1 (adult) 1500 mg/kg, Mig +		
Maximum % change from baseline (cholesterol precursor and metabolites); maximum concentration observed for HP-ß-CD in CSF during initial infusion cycle		
	Initial Infusion Cycle⁎	Full Treatment Period
Lathosterol	−22.9	−26.8
4β-HC	155.0	155.0
27-HC	187.6	187.6
24S-HC	117.2	117.2
Max CSF HP-β-CD (μM)	25.1	


Parameters assessed at baseline, and at 12, 24, 36, and 48 weeks						
	Units	Baseline	W12	W24	W36	W48
PPCS	ng/mL	2.5	0.7	1.0	Wdn	
17D-NPC-CSS total	Score	29	30	35	Wdn	
CGI-I	Score	3	4	6	Wdn	
SARA- Gait	Score	8	8	8	Wdn	
Speech disturbance	Score	2	2	2	Wdn	
Liver size	cm	12.2	14.6	13	Wdn	
Spleen size	cm	13.5	13	12.5	Wdn	

Pt 2 (adult) 2500 mg/kg, Mig +		
Maximum % change from baseline (cholesterol precursor and metabolites); maximum concentration observed for HP-ß-CD in CSF during initial infusion cycle		
	Initial Infusion Cycle⁎	Full Treatment Period
Lathosterol	−46.6	−46.6
4β-HC	228.5	228.5
27-HC	299.9	299.9
24S-HC	132.7	132.7
Max CSF HP-β-CD (μM)	241.1	


Parameters assessed at baseline, and at 12, 24, 36, and 48 weeks						
	Units	Baseline	W12	W24	W36	W48
PPCS	ng/mL	13.7	8.1	6.2	6.5	6.8
17D-NPC-CSS total	Score	24	22	22	19	21
CGI-I	Score	3	4	3	3	3
SARA- Gait	Score	5	4	5	4	4
Speech disturbance	Score	3	2	3	2	3
Liver size	cm	16.1	16.3	16.5	16.6	16.3
Spleen size	cm	17	17	17	17.7	16.6

Pt 3 (adult) 2000 mg/kg, Mig +		
Maximum % change from baseline (cholesterol precursor and metabolites); maximum concentration observed for HP-ß-CD in CSF during initial infusion cycle		
	Initial Infusion Cycle⁎	Full Treatment Period
Lathosterol	−48.3	−59.1
4β-HC	134.6	134.6
27-HC	197.7	197.7
24S-HC	116.1	116.1
Max CSF HP-β-CD (μM)	17.0	


Parameters assessed at baseline, and at 12, 24, 36, and 48 weeks						
	Units	Baseline	W12	W24	W36	W48
PPCS	ng/mL	4.4	2.1	2.7	2.1	2.3
17D-NPC-CSS total	Score	12	12	12	12	9
CGI-I	Score	4	3	3	3	3
SARA- Gait	Score	2	1	1	2	1
Speech disturbance	Score	2	2	2	2	2
Liver size	cm	15	14.6	14.5	ND	15.0
Spleen size	cm	14.5	13.5	12.4	13.4	12.7

Pt 4 (pediatric) 1500 mg/kg, Mig +		
Maximum % change from baseline (cholesterol precursor and metabolites); maximum concentration observed for HP-ß-CD in CSF during initial infusion cycle		
	Initial Infusion Cycle⁎	Full Treatment Period
Lathosterol	ND	45.4
4β-HC	ND	188.4
27-HC	ND	194.4
24S-HC	ND	143.8
Max CSF HP-β-CD (μM)	303.4	


Parameters assessed at baseline, and at 12, 24, 36, and 48 weeks						
	Units	Baseline	W12	W24	W36	W48
PPCS	ng/mL	3.7	2.2†	ND	ND	ND
17D-NPC-CSS total	Score	23	ND	ND	ND	ND
CGI-I	Score	1	ND	ND	ND	ND
SARA- Gait	Score	5	ND	ND	ND	ND
Speech disturbance	Score	6	ND	ND	ND	ND
Liver size	cm	12.3	ND	ND	ND	ND
Spleen size	cm	10.3	ND	ND	ND	ND

Pt 5 (pediatric) 1500 mg/kg, Mig +		
Maximum % change from baseline (cholesterol precursor and metabolites); maximum concentration observed for HP-β-CD in CSF during initial infusion cycle		
	Initial Infusion Cycle⁎	Full Treatment Period
Lathosterol	ND	−40.7
4β-HC	ND	161.9
27-HC	ND	231.4
24S-HC	ND	132.1
Max CSF HP-β-CD (μM)	ND	


Parameters assessed at baseline, and at 12, 24, 36, and 48 weeks						
	Units	Baseline	W12	W24	W36	W48
PPCS	ng/mL	8.1	2.6	2.9†	2.4	1.9
17D-NPC-CSS total	Score	24	23	23	23	22
CGI-I	Score	4	3	4	3	3
SARA- Gait	Score	ND	ND	ND	ND	ND
Speech disturbance	Score	ND	ND	ND	ND	ND
Liver size	cm	7.3	7.2	6.2	7	9
Spleen size	cm	13.2	ND	10.3‡	9.5‡	9.0

Pt 6 (pediatric) 2000 mg/kg		
Maximum % change from baseline (cholesterol precursor and metabolites); maximum concentration observed for HP-β-CD in CSF during initial infusion cycle		
	Initial Infusion Cycle⁎	Full Treatment Period
Lathosterol	ND	−76.6
4β-HC	ND	227.7
27-HC	ND	250.5
24S-HC	ND	147.5
Max CSF HP-β-CD (μM)	33.4	


Parameters assessed at baseline, and at 12, 24, 36, and 48 weeks						
	Units	Baseline	W12	W24	W36	W48
PPCS	ng/mL	5.5	1.4†	1.6†	1.5†	2.1†
17D-NPC-CSS total	Score	26	28	15	18	22
CGI-I	Score	2	2	3	3	3
SARA- Gait	Score	2	3	2	2	2
Speech disturbance	Score	2	3	3	3	3
Liver size	cm	12	ND	ND	ND	ND
Spleen size	cm	13.7	14	ND	ND	13.5

Pt 7 (pediatric) 1500 mg/kg, Mig +		
Maximum % change from baseline (cholesterol precursor and metabolites); maximum concentration observed for HP-β-CD in CSF during initial infusion cycle		
	Initial Infusion Cycle⁎	Full Treatment Period
Lathosterol	ND	−48.8
4β-HC	ND	110.2
27-HC	ND	145.7
24S-HC	ND	116.9
Max CSF HP-β-CD (μM)	ND	


Parameters assessed at baseline, and at 12, 24, 36, and 48 weeks						
	Units	Baseline	W12	W24	W36	W48
PPCS	ng/mL	ND	ND	ND	ND	ND
17D-NPC-CSS total	Score	5	5	10	4	3
CGI-I	Score	3	2	2	1	1
SARA- Gait	Score	8	ND	7	6	5
Speech disturbance	Score	5	ND	5	5	4
Liver size	cm	ND	ND	13	9.2	10.5
Spleen size	cm	ND	12.6	13.6	12	12.3

Pt 8 (pediatric) 2500 mg/kg, Mig +		
Maximum % change from baseline (cholesterol precursor and metabolites); maximum concentration observed for HP-ß-CD in CSF during initial infusion cycle		
	Initial Infusion Cycle⁎	Full Treatment Period
Lathosterol	ND	1.1†
4β-HC	ND	165.1
27-HC	ND	199.2
24S-HC	ND	165.6
Max CSF HP-β-CD (μM)	35.3	


Parameters assessed at baseline, and at 12, 24, 36, and 48 weeks						
	Units	Baseline	W12	W24	W36	W48
PPCS	ng/mL	ND	ND	ND	ND	ND
17D-NPC-CSS total	Score	16	14	13	19	17
CGI-I	Score	2	2	3	5	4
SARA- Gait	Score	8	6	6	8	8
Speech disturbance	Score	2	0	0	0	0
Liver size	cm	11	9	9.4	11	9.5
Spleen size	cm	14.4	12.4	13	12.8	13.4

Pt 9 (pediatric) 1500 mg/kg, Mig +		
Maximum % change from baseline (cholesterol precursor and metabolites); maximum concentration observed for HP-ß-CD in CSF during initial infusion cycle		
	Initial Infusion Cycle⁎	Full Treatment Period
Lathosterol	ND	−43.2
4β-HC	ND	152.7
27-HC	ND	169.6
24S-HC	ND	124.5
Max CSF HP-β-CD (μM)	ND	

Parameters assessed at baseline, and at 12, 24, 36, and 48 weeks						
	Units	Baseline	W12	W24	W36	W48
PPCS	ng/mL	ND	ND	ND	ND	Wdn
17D-NPC-CSS total	Score	24	27	38	32	Wdn
CGI-I	Score	4	4	5	5	Wdn
SARA- Gait	Score	7	7	8	6	Wdn
Speech disturbance	Score	5	6	6	6	Wdn
Liver size	cm	12.2	13.5	11.4	14	Wdn
Spleen size	cm	14.5	13.5	13.3	12.5	Wdn

Pt 10 (pediatric) 2000 mg/kg, Mig +		
Maximum % change from baseline (cholesterol precursor and metabolites); maximum concentration observed for HP-ß-CD in CSF during initial infusion cycle		
	Initial Infusion Cycle⁎	Full Treatment Period
Lathosterol	ND	−40.5
4β-HC	ND	142.5
27-HC	ND	232.4
24S-HC	ND	113.5
Max CSF HP-β-CD (μM)	13.1	


Parameters assessed at baseline, and at 12, 24, 36, and 48 weeks						
	Units	Baseline	W12	W24	W36	W48
PPCS	ng/mL	17.7	12.1	11.2	11.6	12.3
17D-NPC-CSS total	Score	15	15	18	18	19
CGI-I	Score	4	3	2	2	2
SARA- Gait	Score	ND	ND	ND	ND	ND
Speech disturbance	Score	ND	ND	ND	ND	ND
Liver size	cm	11.6	11.8	11.8	10.8	10
Spleen size	cm	18.4	17.4	18.5	18	17.5

Pt 11 (pediatric) 2000 mg/kg, Mig +		
Maximum % change from baseline (cholesterol precursor and metabolites); maximum concentration observed for HP-ß-CD in CSF during initial infusion cycle		
	Initial Infusion Cycle⁎	Full Treatment Period
Lathosterol	ND	−16.6
4β-HC	ND	110.2
27-HC	ND	115.1
24S-HC	ND	109.6
Max CSF HP-β-CD (μM)	ND	


Parameters assessed at baseline, and at 12, 24, 36, and 48 weeks						
	Units	Baseline	W12	W24	W36	W48
PPCS	ng/mL	ND	ND	ND	ND	ND
17D-NPC-CSS total	Score	9	5	8	9	8
CGI-I	Score	3	2	3	2	2
SARA- Gait	Score	3	3	3	3	3
Speech disturbance	Score	5	5	4	4	3
Liver size	cm	ND	11	11.7	11.6	11
Spleen size	cm	11.5	13	14.8	13	12

Pt 12 (pediatric) 2500 mg/kg		
Maximum % change from baseline (cholesterol precursor and metabolites); maximum concentration observed for HP-ß-CD in CSF during initial infusion cycle		
	Initial Infusion Cycle⁎	Full Treatment Period
Lathosterol	−45.1	−45.1
4β-HC	192.8	192.8
27-HC	223.3	223.3
24S-HC	140.6	140.6
Max CSF HP-β-CD (μM)	11.5	
		

Parameters assessed at baseline, and at 12, 24, 36, and 48 weeks						
	Units	Baseline	W12	W24	W36	W48
PPCS	ng/mL	2.3	0.5	0.5	0.7	0.8
17D-NPC-CSS total	Score	13	19	18	17	16
CGI-I	Score	2	4	4	ND	4
SARA- Gait	Score	1	2	2	ND	1
Speech disturbance	Score	1	1	1	ND	2
Liver size	cm	12	11.8	13	12.5	12
Spleen size	cm	10.4	9.7	9.5	10	9.4

⁎ Patient 1 had a complete dataset for the initial infusion cycle.

⁎ Patient 2 had a complete dataset for the initial infusion cycle.

⁎ Patient 3 had a complete dataset for the initial infusion cycle.

⁎ Patient 4 had an incomplete dataset for the initial infusion cycle.

† PPCS value was missing at this visit and was replaced by the value obtained at the following visit.

⁎ Patient 5 had an incomplete dataset for the initial infusion cycle.

† PPCS value was missing at this visit and was replaced by the value obtained at the following visit.

‡ W24 done at W25 and W36 done at W37.

⁎ Patient 6 had an incomplete dataset for the initial infusion cycle.

† PPCS value was missing at this visit and was replaced by the value obtained at the following visit.

⁎ Patient 7 had an incomplete dataset for the initial infusion cycle.

⁎ Patient 8 had an incomplete dataset for the initial infusion cycle.

† Lathosterol values trended upward from baseline for this patient; the maximum percentage change from baseline for lathosterol for the full treatment period is a positive value of 1.1%.

⁎ Patient 9 had an incomplete dataset for the initial infusion cycle.

⁎ Patient 10 had an incomplete dataset for the initial infusion cycle.

⁎ Patient 11 had an incomplete dataset for the initial infusion cycle.

⁎ Patient 12 had a complete dataset for the initial infusion cycle.

**Table:** 

				Screening		Week 12		Week 24		Week 36		Week 48	
ID	Age	Sex	Dose group	Size (cm)	Assessment	Size (cm)	Assessment	Size (cm)	Assessment	Size (cm)	Assessment	Size (cm)	Assessment
1	34	F	1500	12.2	Normal	14.6	Normal	13	Normal	ND	ND	ND	ND
2	21	F	2500	16.1	Enlarged	16.3	Enlarged	16.5	Enlarged	16.6	Enlarged	16.3	Enlarged
3	39	F	2000	15	Upper Normal	14.6	Normal	14.5	Normal	ND	ND	15	Upper Normal
4	15	F	1500	12.3	Normal	ND	ND	ND	ND	ND	ND	ND	ND
5	4	M	1500	7.3	Normal	7.2	Normal	6.2	Normal	7	Normal	9	Normal
6	11	M	2000	12	Normal	ND	ND	ND	ND	ND	ND	ND	ND
7	2	F	1500	ND	ND	ND	ND	13	Enlarged	9.2	Normal	10.5	Upper Normal
8	3	M	2500	11	Normal	9	Normal	9.4	Normal	11	Normal	9.5	Normal
9	6	M	1500	12.2	Normal	`13.5	Mildly Enlarged	11.4	Normal	14	Mildly Enlarged	ND	ND
10	2	M	2000	11.6	Enlarged	11.8	Enlarged	11.8	Enlarged	10.8	Upper Normal	10	Normal
11	2	M	2000	ND	ND	11	Borderline Enlarged	11.7	Enlarged	11.6	Enlarged	11	Borderline Enlarged
12	8	M	2500	12	Normal	11.8	Normal	13	Normal	12.5	Normal	12	Normal

ND – not determined.

Reference ranges used were the age-based liver size charts as published online: https://radiologyassistant.nl/pediatrics/normal-values/normal-values-ultrasound.

**Normal:** Within normal range.

**Upper normal**^**a**^: Organ size measurement is equivalent or + 0.5 cm above the highest number for the matching age.

**Borderline enlarged**^**b**^: Organ size is between 0.6 and 1 cm above the highest number for the matching age.

**Enlarged:** >1 cm above the upper limit of normal.

^a^Example**:** Upper normal is 12 cm on the list, and the patient's measurement is 12–12.5 cm.

^b^Example: Upper normal is 12 cm on the list, and the patient's measurement is 12.6–13 cm.

References: neonates [57] and children [58].

**Table:** 

				Screening		Week 12		Week 24		Week 36		Week 48	
ID	Age	Sex	Dose group	Size (cm)	Assessment	Size (cm)	Assessment	Size (cm)	Assessment	Size (cm)	Assessment	Size (cm)	Assessment
1	34	F	1500	13.5	Enlarged	13	Enlarged	12.5	Normal	ND	ND	ND	ND
2	21	F	2500	17	Enlarged	17	Enlarged	17	Enlarged	17.7	Enlarged	16.6	Enlarged
3	39	F	2000	14.5	Enlarged	13.5	Enlarged	12.4	Normal	13.4	Enlarged	12.7	Borderline Enlarged
4	15	F	1500	10.3	Normal	ND	ND	ND	ND	ND	ND	ND	ND
5	4	M	1500	13.2	Enlarged	ND	ND	10.3	Borderline Enlarged	9.5	Normal	9	Normal
6	11	M	2000	13.7	Enlarged	14	Enlarged	ND	ND	ND	ND	13.5	Enlarged
7	2	F	1500	ND	ND	12.6	Enlarged	13.6	Enlarged	12	Enlarged	12.3	Enlarged
8	3	M	2500	14.4	Enlarged	12.4	Enlarged	13	Enlarged	12.8	Enlarged	13.4	Enlarged
9	6	M	1500	14.5	Enlarged	13.5	Enlarged	13.3	Enlarged	12.5	Enlarged	ND	ND
10	2	M	2000	18.4	Enlarged	17.4	Enlarged	18.5	Enlarged	18	Enlarged	17.5	Enlarged
11	2	M	2000	11.5	Enlarged	13	Enlarged	14.8	Enlarged	13	Enlarged	12	Enlarged
12	8	M	2500	10.4	Normal	9.7	Normal	9.5	Normal	10	Normal	9.4	Normal

ND – not determined.

Reference ranges used were the age-based spleen size charts as published online: https://radiologyassistant.nl/pediatrics/normal-values/normal-values-ultrasound.

**Normal:** Within normal range.

**Upper normal**^**a**^: Organ size measurement is equivalent or + 0.5 cm above the highest number for the matching age.

**Borderline enlarged**^**b**^: Organ size is between 0.6 and 1 cm above the highest number for the matching age.

**Enlarged:** >1 cm above the upper limit of normal.

^a^Example**:** Upper normal is 12 cm on the list, and the patient's measurement is 12–12.5 cm.

^b^Example: Upper normal is 12 cm on the list, and the patient's measurement is 12.6–13 cm.

References: neonates [57] and children [59].

**Table:** 

Number of Patients (rating at Week 48 compared with Baseline)	Clinician Global Impression of Improvement (CGI—I) Scale	Score
0	Very much worse	7
0	Much worse	6
0	Minimally worse	5
2	No change	4
5	Minimally improved	3
1	Much improved	2
1	Very much improved	1
3⁎	Not assessed	0

Number of Patients (rating at Week 48 compared with Baseline)	Patient Global Impression of Change (PGIC) Scale	Score
0	No change (or worse)	1
0	Almost the same, hardly any change at all	2
2	A little better but no noticeable change	3
1	Somewhat better but the change has not made any real difference	4
4	Moderately better and a slight but noticeable change	5
1	Better and a definite improvement that has made a real and worthwhile difference	6
1	A great deal better and a considerable improvement that has made all the difference	7

⁎ Patient 1, withdrawn at Week 24; Patient 4, parent refused consent; Patient 9, missed Week 48 because of COVID-19 pandemic restrictions.

**Table:** 

TEAE reported system organ class and preferred term	1500 mg/kg	No. Events as % of Total No. Infusions	2000 mg/kg	No. Events as % of Total No. Infusions	2500 mg/kg	No. Events as % of Total No. Infusions	Total of All TEAEs	All TEAEs as % of Total No. Infusions
	(N = 5)		(N = 4)		(N = 3)		(N = 12)	
	n		n		n		n	
Infections & Infestations								
Upper respiratory tract infection	4	7.8	1	4.0	3	6.0	7	5.6
Nasopharyngitis	1	4.0	2	8.0	3	6.1	6	6.1
Rhinitis	2	4.2	4	14.8	6	24.0	12	12.1
Tonsillitis	0	0.0	1	3.7	1	3.8	2	3.8
Viral Infection	1	4.5	1	4.3	0	0.0	2	4.4
Gastrointestinal Disorders								
Vomiting	3	30.0	4	5.5	6	11.8	11	8.2
Diarrhea	0	0.0	3	12.0	6	8.0	9	9.0
Nausea	0	0.0	4	7.7	0	0.0	2	7.7
Nervous System Disorders								
Seizure	10	26.3	2	8.0	4	16.7	16	18.4
Cataplexy	2	20.0	0	0.0	2	8.0	4	11.4
Cerebrospinal fluid leakage	1	4.5	1	4.0	0	0.0	2	4.3
General Disorders and Administration Site Conditions								
Pyrexia	1	4.5	0	0.0	4	5.3	5	5.2
Fatigue	0	0.0	1	4.0	1	4.0	2	4.0
Peripheral swelling	0	0.0	1	4.3	1	4.0	2	4.2
Injury, Poisoning and Procedural Complications14.73								
Contusion	0	0.0	1	4.0	3	12.0	4	8.0
Respiratory, Thoracic and Mediastinal Disorders								
Cough	3	5.3	2	8.0	7	14.3	12	9.1
Rhinorrhea	1	4.0	1	4.0	0	0.0	2	4.0
Ear and Labyrinth Disorders								
Hypoacusis	1	5.3	2	3.8	0	0.0	3	4.2
Musculoskeletal and Connective Tissue Disorders								
Back pain	0	0.0	2	4.2	0	0.0	2	4.2
Skin and Subcutaneous Tissue Disorders								
Rash	4	40.0	1	4.0	0	0.0	5	14.3
Renal and Urinary Disorders								
Incontinence	0	0.0	1	4.0	1	4.0	2	4.0
**Serious TEAE in any group**	8	42.1	2	8	4	7.8	14	14.7

TEAE, treatment-emergent adverse event.

TEAEs were coded using MedDRA Version 19.1.

## Data Availability

Data will be made available on request.

## Appendix A Appendix

### A.1 Patient panels

Abbreviations for the Patient Panels: 4β-HC – 4β-hydroxycholesterol; 27-HC – 27-hydroxycholesterol; 24S-HC – 24S-hydroxycholesterol; 17D-NPC-CSS – 17-Domain Niemann-Pick disease Type C-Clinical Severity Scale; CGI-I – Clinical Global Impression of Improvement; CSF – cerebrospinal fluid; HP-β-CD – hydroxypropyl-β-cyclodextrin; Mig – miglustat; ND – not determined; PPCS – *N*-palmitoyl-*O*-phosphocholineserine; SARA – Scale for the Assessment and Rating of Ataxia; Wdn – withdrawn.

The lathosterol, 4β-hydroxycholesterol, 27-hydroxycholesterol and 24S-hydroxycholesterol data in the Patient Panels are the maximum percentage change from baseline during the initial infusion cycle (for patients with a complete set of measurements during this infusion cycle) and the maximum percentage change from baseline determined during the entire 48-week period. Lathosterol is a cholesterol precursor and therefore has a negative maximum percentage change from baseline; 4β-hydroxycholesterol, 27-hydroxycholesterol, and 24S-hydroxycholesterol are cholesterol metabolites and therefore have a positive maximum percentage change from baseline.

## References

[1] Geberhiwot T., Moro A., Dardis A. (2018). Consensus clinical management guidelines for Niemann-Pick disease type C. Orphanet. J. Rare Dis..

[2] Wassif C.A., Cross J.L., Iben J. (2016). High incidence of unrecognized visceral/neurological late-onset Niemann-pick disease, type C1, predicted by analysis of massively parallel sequencing data sets. Genet. Med..

[3] Patterson M.C., Clayton P., Gissen P. (2017). Recommendations for the detection and diagnosis of Niemann-pick disease type C: an update. Neurol. Clin. Pract..

[4] Vanier M.T. (2010). Niemann-pick disease type C. Orphanet. J. Rare Dis..

[5] Yamamoto T., Nanba E., Ninomiya H. (1999). NPC1 gene mutations in Japanese patients with Niemann-pick disease type C. Hum. Genet..

[6] Greer W.L., Dobson M.J., Girouard G.S. (1999). Mutations in NPC1 highlight a conserved NPC1-specific cysteine-rich domain. Am. J. Hum. Genet..

[7] Park W.D., O’Brien J.F., Lundquist P.A. (2003). Identification of 58 novel mutations in Niemann-pick disease type C: correlation with biochemical phenotype and importance of PTC1-like domains in NPC1. Hum. Mutat..

[8] NP-C Guidelines Working Group, Wraith J.E., Baumgartner M.R. (2009). Recommendations on the diagnosis and management of Niemann-Pick disease type C. Mol. Genet. Metab..

[9] Hammond N., Munkacsi A.B., Sturley S.L. (1864). The complexity of a monogenic neurodegenerative disease: more than two decades of therapeutic driven research into Niemann-pick type C disease. Biochim. Biophys. Acta Mol. Cell Biol. Lipids.

[10] Newton J., Milstien S., Spiegel S. (2018). Niemann-pick type C disease: the atypical sphingolipidosis. Adv. Biol. Regul..

[11] Wheeler S., Sillence D.J. (2020). Niemann-pick type C disease: cellular pathology and pharmacotherapy. J. Neurochem..

[12] Walkley S.U., Vanier M.T. (2009). Secondary lipid accumulation in lysosomal disease. Biochim. Biophys. Acta.

[13] Sarna J.R., Larouche M., Marzban H., Sillitoe R.V., Rancourt D.E., Hawkes R. (2003). Patterned Purkinje cell degeneration in mouse models of Niemann-pick type C disease. J. Comp. Neurol..

[14] Davidson C.D., Ali N.F., Micsenyi M.C. (2009). Chronic cyclodextrin treatment of murine Niemann-pick C disease ameliorates neuronal cholesterol and glycosphingolipid storage and disease progression. PLoS One.

[15] Abel L.A., Walterfang M., Fietz M., Bowman E.A., Velakoulis D. (2009). Saccades in adult Niemann-pick disease type C reflect frontal, brainstem, and biochemical deficits. Neurology.

[16] Sévin M., Lesca G., Baumann N. (2007). The adult form of Niemann-pick disease type C. Brain.

[17] Schicks J., Müller Vom Hagen J., Bauer P. (2013). Niemann-pick type C is frequent in adult ataxia with cognitive decline and vertical gaze palsy. Neurology.

[18] Battisti C., Tarugi P., Dotti M.T. (2003). Adult-onset Niemann-pick type C disease: a clinical, neuroimaging, and molecular genetic study. Mov. Disord..

[19] Cariati I., Masuelli L., Bei R., Tancredi V., Frank C., D’Arcangelo G. (2021). Neurodegeneration in Niemann-pick type C disease: an updated review on pharmacological and non-pharmacological approaches to counteract brain and cognitive impairment. Int. J. Mol. Sci..

[20] Trendelenburg G., Vanier M.T., Maza S. (2006). Niemann-pick type C disease in a 68-year-old patient. J. Neurol. Neurosurg. Psychiatry.

[21] Patterson M.C., Vecchio D., Prady H., Abel L., Wraith J.E. (2007). Miglustat for treatment of Niemann-pick C disease: a randomised controlled study. Lancet Neurol..

[22] Patterson M.C., Garver W.S., Giugliani R. (2020). Long-term survival outcomes of patients with Niemann-pick disease type C receiving miglustat treatment: a large retrospective observational study. J. Inherit. Metab. Dis..

[23] emc (2023). Miglustat 100mg Hard Capsules - Summary of Product Characteristics (SmPC) - (emc) (medicines.org.uk). https://www.medicines.org.uk/emc/product/39/smpc.

[24] Belmatoug N., Burlina A., Giraldo P. (2011). Gastrointestinal disturbances and their management in miglustat-treated patients. J. Inherit. Metab. Dis..

[25] Karimzadeh P., Tonekaboni S.H., Ashrafi M.R. (2013). Effects of miglustat on stabilization of neurological disorder in niemann-pick disease type C: Iranian pediatric case series. J. Child Neurol..

[26] Brewster M.E., Loftsson T. (2007). Cyclodextrins as pharmaceutical solubilizers. Adv. Drug Deliv. Rev..

[27] Loftsson T., Brewster M.E. (2012). Cyclodextrins as functional excipients: methods to enhance complexation efficiency. J. Pharm. Sci..

[28] Liu B., Li H., Repa J.J., Turley S.D., Dietschy J.M. (2008). Genetic variations and treatments that affect the lifespan of the NPC1 mouse. J. Lipid Res..

[29] Liu B., Turley S.D., Burns D.K., Miller A.M., Repa J.J., Dietschy J.M. (2009). Reversal of defective lysosomal transport in NPC disease ameliorates liver dysfunction and neurodegeneration in the npc1−/− mouse. Proc. Natl. Acad. Sci. U. S. A..

[30] Liu B. (2012). Therapeutic potential of cyclodextrins in the treatment of Niemann-pick type C disease. Clin. Lipidol..

[31] Camargo F., Erickson R.P., Garver W.S. (2001). Cyclodextrins in the treatment of a mouse model of Niemann-pick C disease. Life Sci..

[32] Ramirez C.M., Liu B., Taylor A.M. (2010). Weekly cyclodextrin administration normalizes cholesterol metabolism in nearly every organ of the Niemann-pick type C1 mouse and markedly prolongs life. Pediatr. Res..

[33] Vite C.H., Bagel J.H., Swain G.P. (2015). Intracisternal cyclodextrin prevents cerebellar dysfunction and Purkinje cell death in feline Niemann-Pick type C1 disease. Sci. Transl. Med..

[34] Kempen H.J., Ganlatz J.F., Gevers Leuven J.A., van der Voort H.A., Katan M.B. (1988). Serum lathosterol concentration is an indicator of whole-body cholesterol synthesis in humans. J. Lipid Res..

[35] Yanjanin N.M., Velez J.I., Gropman A. (2010). Linear clinical progression, independent of age of onset, in Niemann-pick disease, type C. Am. J. Med. Genet. B Neuropsychiatr. Genet..

[36] Te Vruchte D., Speak A.O., Wallom K. (2014). Relative acidic compartment volume as a lysosomal storage disorder–associated biomarker. J. Clin. Invest..

[37] Riestra-Candelaria B.L., Rodríguez-Mojica W., Vázquez-Quiñones L.E., Jorge J.C. (2016). Ultrasound accuracy of liver length measurement with cadaveric specimens. J. Diagn. Med. Sonogr..

[38] OHSU (2023). http://www.ohsu.edu/xd/education/schools/school-of-medicine/departments/clinical-departments/diagnostic-radiology/pediatric-radiology-normal-measurements/gastrointestinal-measurements.cfm#spleen.

[39] Roussounis S.H., Gaussen T.H., Stratton P. (1987). A 2-year follow-up study of children with motor coordination problems identified at school entry age. Child Care Health Dev..

[40] Kamper S.J., Maher C.G., Mackay G. (2009). Global rating of change scales: a review of strengths and weaknesses and considerations for design. J. Man. Manip. Ther..

[41] Eremenco S., Chen W.H., Blum S.I. (2022). Comparing patient global impression of severity and patient global impression of change to evaluate test–retest reliability of depression, non-small cell lung cancer, and asthma measures. Qual. Life Res..

[42] Kantner I., Erben R.G. (2012). Long-term parenteral administration of 2-hydroxypropyl-β-cyclodextrin causes bone loss. Toxicol. Pathol..

[43] Mattsson N., Zteerbuergh H., Bianconi S. (2012). Miglustat treatment may reduce cerebrospinal fluid levels of the axonal degeneration marker tau in Niemann-pick type C. JIMD Rep..

[44] (March 18, 2019). Meeting Report for: Niemann-Pick Type C Patient and Caregiver Voices: Externally-Led, Patient-Focused Drug Development Meeting with the U.S. Food and Drug Administration (FDA).

[45] Hastings C., Liu B., Hurst B. (2022). Intravenous 2-hydroxypropyl-β-cyclodextrin (Trappsol® CycloTM) demonstrates biological activity and impacts cholesterol metabolism in the central nervous system and peripheral tissues in adult subjects with Niemann-pick disease type C1: results of a phase 1 trial. Mol. Genet. Metab..

[46] Hastings C., Vieira C., Liu B. (2019). Expanded access with intravenous hydroxypropyl-beta-cyclodextrin to treat children and young adults with Niemann-Pick disease type C1: a case report analysis. Orphanet. J. Rare Dis..

[47] Maarup T.J., Chen A.H., Porter F.D. (2015). Intrathecal 2-hydroxypropyl-beta-cyclodextrin in a single patient with Niemann-Pick C1. Mol. Genet. Metab..

[48] Ory D.S., Ottinger E.A., Farhat N.Y. (2017). Intrathecal 2-hydroxypropyl-beta-cyclodextrin decreases neurological disease progression in Niemann-Pick disease, type C1: a non-randomised, open-label, phase 1-2 trial. Lancet.

[49] Liu X., Ding D., Chen G.D. (2020). 2-Hydroxypropyl-β-cyclodextrin ototoxicity in adult rats: rapid onset and massive destruction of both inner and outer hair cells above a critical dose. Neurotox. Res..

[50] Mengel E., Patterson M.C., Da Riol R.M. (2021). Efficacy and safety of arimoclomol in Niemann-Pick disease type C: results from a double-blind, randomised, placebo-controlled, multinational phase 2/3 trial of a novel treatment. J. Inherit. Metab. Dis..

[51] Björkhem I., Lütjohann D., Breuer O., Sakinis A., Wennmalm A. (1997). Importance of a novel oxidative mechanism for elimination of brain cholesterol. Turnover of cholesterol and 24(S)-hydroxycholesterol in rat brain as measured with 18O2 techniques in vivo and in vitro. J. Biol. Chem..

[52] Shim K.H., Kang M.J., Suh J.W. (2020). CSF total tau/α-synuclein ratio improved the diagnostic performance for Alzheimer's disease as an indicator of tau phosphorylation. Alzheimers Res. Ther..

[53] Okamura N., Arai H., Maruyama M. (2002). Combined analysis of CSF tau levels and [(123)I]iodoamphetamine SPECT in mild cognitive impairment: implications for a novel predictor of Alzheimer’s disease. Am. J. Psychiatry.

[54] Giese A.K., Mascher H., Grittner U. (2015). A novel, highly sensitive and specific biomarker for Niemann-pick type C1 disease. Orphanet. J. Rare Dis..

[55] Breilyn M.S., Zhang W., Yu C., Wasserstein M.P. (2021). Plasma lyso-sphingomyelin levels are positively associated with clinical severity in acid sphingomyelinase deficiency. Mol. Genet. Metab. Rep..

[56] Sidhu R., Kell P., Dietzen D.J. (2020). Application of N-palmitoyl-O-phosphocholineserine for diagnosis and assessment of response to treatment in Niemann-pick type C disease. Mol. Genet. Metab..

[57] Soyupak S.K., Narli N., Yapicioğlu H., Satar M., Aksungur E.H. (2002). Sonographic measurements of the liver, spleen and kidney dimensions in the healthy term and preterm newborns. Eur. J. Radiol..

[58] Konuş O.L., Ozdemir A., Akkaya A., Erbaş G., Celik H., Işik S. (1998). Normal liver, spleen, and kidney dimensions in neonates, infants, and children: evaluation with sonography. AJR Am. J. Roentgenol..

[59] Megremis S.D., Vlachonikolis I.G., Tsilimigaki A.M. (2004). Spleen length in childhood with US: normal values based on age, sex, and somatometric parameters. Radiology..

